# A novel *Erwiniaceae* gut symbiont modulates gene expression of the intracellular bacterium *Cardinium* in the stored product mite *Tyrophagus putrescentiae*

**DOI:** 10.1128/msphere.00879-24

**Published:** 2025-03-24

**Authors:** Jan Hubert, Eliza Glowska-Patyniak, Scot E. Dowd, Pavel B. Klimov

**Affiliations:** 1 Czech Agrifood Research Center, Prague, Czechia; 2 Department of Animal Morphology, Faculty of Biology, Adam Mickiewicz University in Poznan, Poznan, Poland; 3 MR DNA (Molecular Research LP), Shallowater, Texas, USA; 4 Purdue University, Lilly Hall of Life Sciences, West Lafayette, Indiana, USA; University of Michigan, Ann Arbor, Michigan, USA

**Keywords:** stored product mite, bacterial symbionts, gene expression, *Cardinium*, *Wolbachia*, *Erwiniaceae*, *Tyrophagus putrescentiae*, allergens, *Sodalis*

## Abstract

We examined host and bacterial gene expression profiles in the stored product mite *Tyrophagus putrescentiae* co-infected with *Wolbachia* (wTPut) and *Cardinium* (cTPut) while varying the presence of the *Erwiniaceae* symbiont (SLS). SLS, a novel symbiont in the family *Erwiniaceae*, with a genome size of 1.7 Mb, is found in 16% of mite species in infected cultures. In addition, SLS was detected in mite feces but not in their eggs. Although *Wolbachia* expression remained unchanged, the presence or absence of SLS significantly affected *Cardinium* expression. It indicated that the effect of *Wolbachia* on SLS was neutral. In SLS-positive samples, *Cardinium* exhibited 29 upregulated and 48 downregulated genes compared to SLS-negative samples. Furthermore, *Cardinium* gene expression strongly correlated with mite KEGG gene expression in SLS-positive samples. Positive Spearman’s correlations between *Cardinium* gene expression and mite KEGG immune and regulatory pathways were doubled in SLS-positive compared to SLS-negative samples. The diversity of expressed genes in the mite host decreased in the presence of SLS. *Cardinium* had more interacting genes to mite host in SLS-positive samples than without SLS. Transposases are the most affected *Cardinium* genes, showing upregulation in the presence of SLS. Correlation analyses revealed interactions between *Cardinium* and SLS via mite immune and regulatory pathways, including lysosome, ubiquitin-mediated proteolysis, PIK3_Akt, and cGMP-PKG. The results showed that *Cardinium* indirectly affects the gut symbionts of mites.

**IMPORTANCE:**

This study introduces a new model to analyze interactions between intracellular bacterial symbionts, gut bacterial symbionts, and their mite hosts. Using gene expression correlations, we investigated how the intracellular *Cardinium* responds to the novel *Erwiniaceae* gut symbiont in the mold mite *Tyrophagus putrescentiae*. The data showed that both mite and *Cardinium* gene expression are different in the samples with and without *Erwiniaceae* symbionts. In the presence of *Erwiniaceae* symbionts, *Cardinium* increased the interaction with the mite host in terms of changes in gene expression. The mite immune and regulatory pathway gene expression is differently correlated to *Cardinium* genes in relation to *Erwiniaceae* symbionts. As a well-known producer of allergens, *T. putrescentiae* physiology and thus its allergen production are influenced by both symbionts, potentially affecting the release of allergens into human environments.

## INTRODUCTION

Intracellular, cytoplasmically inherited bacteria, such as *Cardinium* and *Wolbachia*, are commonly found in the reproductive tissues of many arthropods (1–3). These bacteria can manipulate host reproduction through mechanisms such as cytoplasmic incompatibility (CI), parthenogenesis induction, male killing, and feminization of genetic males, although they belong to evolutionary distinct lineages (4–10). *Cardinium* has been detected in approximately 6%–10% of arthropod species (11) and has been reported in 45 mite species across three orders and 14 families (12). The *Cardinium* genome contains complete pathways for biotin and lipoate synthesis, nutrients important for host nutrition (13). However, it remains unclear whether the host utilizes these metabolic products (14).

*Cardinium* associated with arthropods does not form a monophyletic group, suggesting diverse evolutionary histories (14). Currently, there are only four sequenced genomes of mite-associated *Cardinium* strains, representing three distinct lineages. These include (i) symbionts of *Brevipalpus californicus* (cBcaIN1: JAACGE01, cBcaIN2: JAACGD01) and *Brevipalpus yothersi* (cByotN1: JAACGG01); (ii) cDFar (CP101107-9 and VMBH01) from the house dust mite *Dermatophagoides farinae* (15, 16), and (iii) cTPut (JANAVR01 and JAZHET01) from stored product mite *Tyrophagus putrescentiae* (16). Notably, the cDFar genome (1.4 Mb) contains two plasmids, which are absent in the smaller cTPut genome (1.05 Mb) (16). Moreover, cDFar is found only in females, while cTPut occurs in both sexes of *T. putrescentiae* (15, 17). cTPut is closely related to cSfur, a *Cardinium* strain from the insect *Sogatella furcifera* (CP022339) (13). According to previous analyses, *Wolbachia* symbiont of *T. putrescentiae* (wTPut) (GIJY0 and JAUEMM01) formed a new lineage with phylogenetically unrelated and ecologically dissimilar gall *mite Fragariocoptes setiger* (NZ_JACVWV01) (18) and this lineage has been classified as supergroup Q (19, 20).

Interactions between intracellular symbionts and members of the gut microbiome are increasingly being recognized (21). For example, *Cardinium* is abundant in various species of *Tetranychus* and influences microbiota composition in *Amphitetranychus viennensis*, *Tetranychus urticae*, and *Tetranychus turkestani*, suggesting its potential role in shaping the microbial community of spider mites; for example, positive correlation between the relative abundance of *Cardinium* and *Pseudomonas*, and negative to *Thermus*, *Acinetobacter*, *Corynebacterium*, and *Anoxybacillus* (22).

A previous study of *T. putrescentiae* showed a negative correlation between cTPut and other associated bacteria (23), including *Wolbachia*, *Bartonella*-like, *Blattabacterium*-like, *Solitalea*-like, and *Erwiniaceae* symbiont previously named *Sodalis*-like symbionts based on 16S RNA analyses (SLS) (24–28). In *S. furcifera*, cSFur, either alone or in combination with *Wolbachia*, affected bacterial microbiota composition and metabolite levels, impacting host fecundity. In the herbivorous insect *Nilaparvata lugens*, *Cardinium* infection increased the density of a midgut bacterium, *Acinetobacter* (29). cSFur infection upregulated amino acid metabolism (arginine biosynthesis), metabolism of cofactors and vitamins (pantothenate and CoA biosynthesis), and translation (aminoacyl-tRNA biosynthesis) (30). However, despite these findings, we still do not fully understand how *Cardinium* manipulates host gut microbiota.

*Tyrophagus putrescentiae*, the stored product mite, is a common pest found in stored food and animal feed (31). It feeds on a variety of plant and animal-based products, including grains, seeds, dried leaves, cheese, dried ham, and sausages, as well as microorganisms (32). In addition to damaging stored food, *T. putrescentiae* contaminates human environments with allergens (33–36). Symbiotic microorganisms have long been hypothesized to contribute to mite nutrition, but direct evidence for this has been limited (37–39). The symbionts manipulated mites in various ways and influenced mite allergen production in the human environment (40).

Previous studies showed differences in *Cardinium* gene expression between two genomes (5L and 5S) in the experiments (*Cardinium + Wolbachia*) using single-infected (5L and 5S) and experimental (5LP, 5LN, 5SP, and 5SN) cultures of *T. putrescentiae* (41). In this study, we aim to explain these differences (i.e., 5L, 5LN, and 5LP versus 5S, 5SN, and 5SP) with the hypotheses: (i) The *Cardinium* genomes c_1_ and c_2_ were different and (ii) there was an influence of another bacterial symbiont, such as the *Erwiniaceae* symbiont (SLS) found in the 5L but not the 5S cultures of *T. putrescentiae* (42). To test the first hypothesis, we conducted metagenomic analyses of our samples. For the second hypothesis, we analyzed expression patterns in SLS-positive and SLS-negative cultures. SLS presence was detected in single mites, in mite population-level samples, samples of mite feces, and the samples of mite egg samples using conventional PCR and quantified PCR with taxa species-specific primers. Finally, previously mite and SLS and cTPut bacterial gene expression in the published samples of meta-transcriptome published were used for correlation-based analyses (41). KEGG-annotated genes in the mite host were utilized to assess the regulatory and metabolic pathways involved in these interactions.

## RESULTS

### *Cardinium* assemblies and differences between 5S and 5L mite cultures

**Fig 1:**
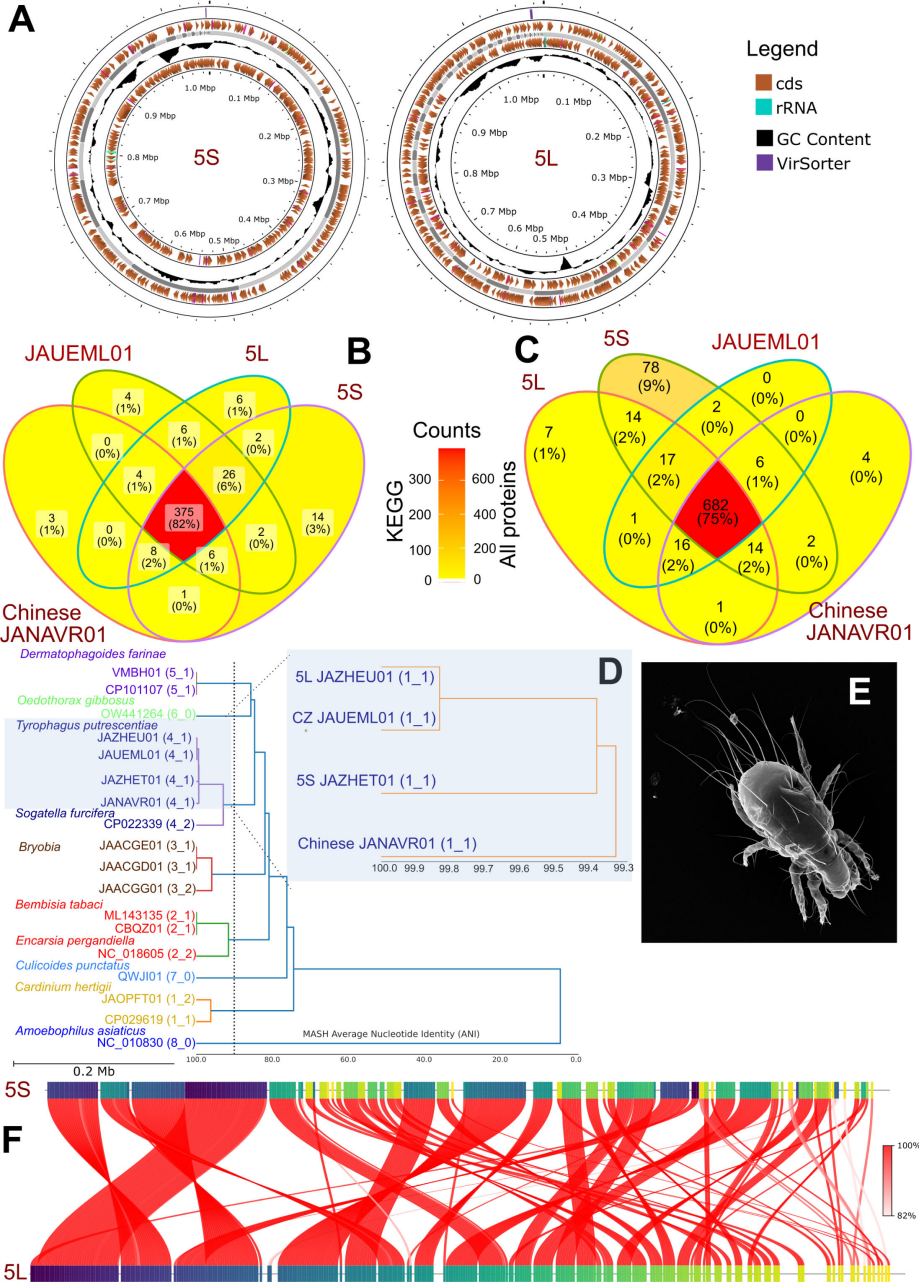
*Cardinium* (cTPut), a bacterial endosymbiont of the stored food mite, *Tyrophagus putrescentiae:* (**A**) Visualization of two *Cardinium* genomes from mite cultures 5L and 5S using PROKSEE; (**B**) Venn diagram comparing *Cardinium* genomes based on KEGG proteins and all predicted proteins (C); (**D**) dREP comparison of known *Cardinium* genomes using average nucleotide identity (ANI); (**E**) scanning electron microscopy (SEM) image of the host, *T. putrescentiae*; (**F**) ANI-based comparison of the two *Cardinium* genomes from cultures 5S and 5L, visualized in PROKSEE.

Two *Cardinium* (cTPut) genomes, JAZHEU01 and JAZHET01 (Fig. 1), were assembled from separate mite cultures, 5S (dried ham, Italy) and 5L (grain, Czechia) (Tables S1 and S2). These genomes have a high identity of GC contents and length with previously sequenced genomes of cTPut from China (JANAVR01) and Czechia (JAUEML01; Table 1); however, the number of all predicted genes and KEGG proteins was slightly higher (Table 1). All genomes (Table S2) had 100% completeness and 92% ANI similarity to the *Cardinium* symbiont of *S. furcifera* (cSFur) (CA_0033519051) (13). All *Cardinium* cTPut genomes shared 82% of predicted proteins and 75% of KEGG proteins. The number of unique proteins decreased from cTPut_5S (JAZHET01; 14 proteins) to cTPut_5L (JAZHEU01; six proteins) (Fig. 1B; Table S3). A MASH ANI-based analysis (43) confirmed that *Cardinium* symbionts form a monophyletic clade of closely related lineages (ANI ranges 78%–96%), with *cSFur* as their sister group. There was a 99% ANI similarity between strains 5L and 5S, supporting the notion that these strains could belong to a single species (Fig. 1D).

**TABLE 1:** Comparison of *Cardinium* (cTPut) and *Erwiniaceae* (SLS) genomes from *Tyrophagus putrescentiae^c^*

Genome	Contigs	GC%	Length	CDS	rRNA	KEGG	Completeness	Contamination	ANI	Matched	Total
China	33	39.4	914,750	750	3	407	100	0	91.89	248	288^*a*^
5L	59	38.5	1,090,917	934	3	446	100	0	92.06	273	334^*a*^
5S	28	38.9	1,051,651	889	3	460	100	0	91.97	288	336^*a*^
CZ	55	38.9	1,051,907	882	3	445	100	0	91.98	273	323^*a*^
SLS	577	52.2	1,806,254	1936	9	1,411	28	4.51	84.16	199	405^*b*^

^*a*^ ANI comparisons were done using a DFAST server, with the reference *Cardinium* (GCA_0033519051).

^*b*^ ANI comparisons were done using a DFAST server, with the reference *Mixta calida* (GCF_002953215).

^*c*^ cTPut genomes: China (JANAVR01); CZ (JAUEML01), 5L (JAZHEU01) and 5S (JAZHET01). SLS (JAVLVR01).cTPut genomes: China (JANAVR01); CZ (JAUEML01), 5L (JAZHEU01) and 5S (JAZHET01). SLS (JAVLVR01).

Distance-based redundancy analysis (dbRDA) was based on *Cardinium* gene expression data (Table S4). The dbRDA separated the samples of two cTPU populations into two different clusters. The clusters separated 5S (JAZHEU01) and 5L (JAZHET01), along the CAP2 axis, this accounted for 9% of the total variation (Table 2, model 1) using the presence/absence of SLS and *Wolbachia* (wTPut) as the factor. The presence of wTPut caused 83% of the variability, as indicated by differences in sample position on the CAP1 axis.

**TABLE 2:** Correlation-based models describing the interaction among *Cardinium* (cTPut), *Wolbachia* (wTPut), and *Erwiniaceae* (SLS) symbionts, and the host mite *Tyrophagus putrescentiae*^*a*^,^*b*^

No.	Dependent variable	Independent variable	Data set	Distance	df	*F*	*R* ^2^
1	cTPut	SLS	Presence	Robust Aitchinson	1	4.723	0.231
		wTPut	Presence		1	7.004	
		Residual			39		
2	cTPut	SLS	Presence	Jaccard	1	5.765	0.460
		wTPut	Presence		1	27.960	
		Residual			39		
3	cTPut sel	SLS	Presence	Robust Aitchinson	1	4.723	0.270
		wTPut	Presence		1	7.004	
		Residual			39		
4	cTPut_(SLS+)	SLS	Expression	Robust Aitchinson	7	2.172	0.539
5	cTPut_sel	SLS	Expression	Robust Aitchinson	9	2.583	0.582
6	cTPut (SLS−)	*Tyrophagus* (SLS−)	Expression	Robust Aitchinson	6	2.116	0.476
7	cTPut (SLS+)	*Tyrophagus* (SLS+)	Expression	Robust Aitchinson	8	2.153	0.589
8	cTPut (SLS+)	*Tyrophagus* and SLS	Expression	Robust Aitchinson	5	1.644	0.535
9	SLS	Mite cultures	Presence	Robust Aitchinson	1	41.500	0.680
		Residual		Robust Aitchinson	19		
10	SLS	wTPut_presence	Presence	Robust Aitchinson	1	0.467	0.154
		Residual		Robust Aitchinson	19		
11	SLS	cTPut (SLS+)	Expression	Robust Aitchinson	5	1.866	0.384
12	SLS	cTPut _sel	Expression	Robust Aitchinson	5	1.947	0.327
13	SLS	*Tyrophagus* (SLS+)	Expression	Robust Aitchinson	5	1.809	0.376
14	SLS	*Tyrophagus* and cTPut (SLS+)	Expression	Robust Aitchinson	6	1.896	0.448
15	*Tyrophagus*	wTPut	Presence	Robust Aitchinson	1	7.653	0.334
		cTPut	Presence		1	13.983	
		SLS	Presence		1	4.356	
		Residual			52		
16	*Tyrophagus* (SLS−)	cTPut (SLS−)	Expression	Robust Aitchinson	6	2.117	0.476
17	*Tyrophagus* (SLS+)	SLS	Expression	Robust Aitchinson	5	2.298	0.434
18	*Tyrophagus* (SLS+)	cTPut (SLS+)	Expression	Robust Aitchinson	4	2.471	0.382
19	*Tyrophagus* (SLS+)	SLS and cTPut (SLS+)	Expression	Robust Aitchinson	4	2.581	0.392
20	*Tyrophagus*_immune (SLS-)	cTPut (SLS-)	Expression	Robust Aitchinson	5	1.838	0.379
21	*Tyrophagus*_immune (SLS+)	cTPut (SLS+)	Expression	Robust Aitchinson	6	2.412	0.508
22	*Tyrophagus*_immune (SLS+)	SLS (SLS+)	Expression	Robust Aitchinson	5	2.718	0.475
23	*Tyrophagus*_immune (SLS+)	cTPut and SLS (SLS+)	Expression	Robust Aitchinson	7	2.4911	0.573

^*a*^ Distance-based redundancy analysis (dbRDA) models were constructed using gene expression data and KEGG-assigned mite genes. All analyses yielded *P*-values below 0.05.

^*b*^ Conventions in variable coding: SLS+ = *Erwiniaceae* symbiont present (5L, 5LP, and 5LN); SLS− = *Erwiniaceae* symbiont absent (5S, 5SP, and 5SN); selected genes (sel) = genes with differential expression identified using false discovery rate (FDR) and *Cardinium* protein outliers (Table S13); immune = KEGG gene expression in selected immune and regulatory pathways; residuals are residual variability in the dbRDA models did not explained by tested environmental variables. The dependent variables included predicted protein expression in all cases, while factors included presence/absence or predicted protein expression.

Gene presence/absence data using the Jaccard distance also identified significant differences in cTPut from the samples with SLS (cultures: 5L, 5LN, and 5LP) and without SLS (cultures: 5S, 5SN, and 5SP) (Fig. S2). However, further detailed analyses revealed that a significant proportion of the proteins responsible for these differences are insertion sequences (IS) or their candidates (Fig. S1). A previous study on *Cardinium* described a novel IS-derived miniature inverted-repeat transposable element (MITE), which is likely being actively maintained by intact copies of its parental transposase (44). In our *Cardinium* species, we also identified MITEs using HMMER (Fig. S2, Table S5), including transposase fragments from IS256, IS4, and IS6. Because many of the transposases are incomplete, we suggested them as an MITE, also. Transposase fragments were consistently identified in all genomes of cTPut (Fig. S1). Overall, our data suggest that the two populations of cTPut from cultures 5S and 5L (JAZHEU01 and JAZHET01) were nearly identical.

### *Erwiniaceae* symbiont genome and transcriptome

The *Erwiniaceae* (SLS) genome (GenBank: JAVLVR01) has a total size of 1,758,788 bp with 434 contigs (N50 = 912 bp), a coverage of 8,507 (PILON for both metagenome and metatranscriptome samples), and a GC content of 52.2% (Table 1, Table S6). The assemblage represents a new species as determined using the MASH algorithm (45) through the Type (Strain) Genome Server (TYGS) at https://tygs.dsmz.de (46, 47) integrated with the List of Prokaryotic Names with Standing in Nomenclature (LPSN; https://lpsn.dsmz.de) (47).

**Fig 2:**
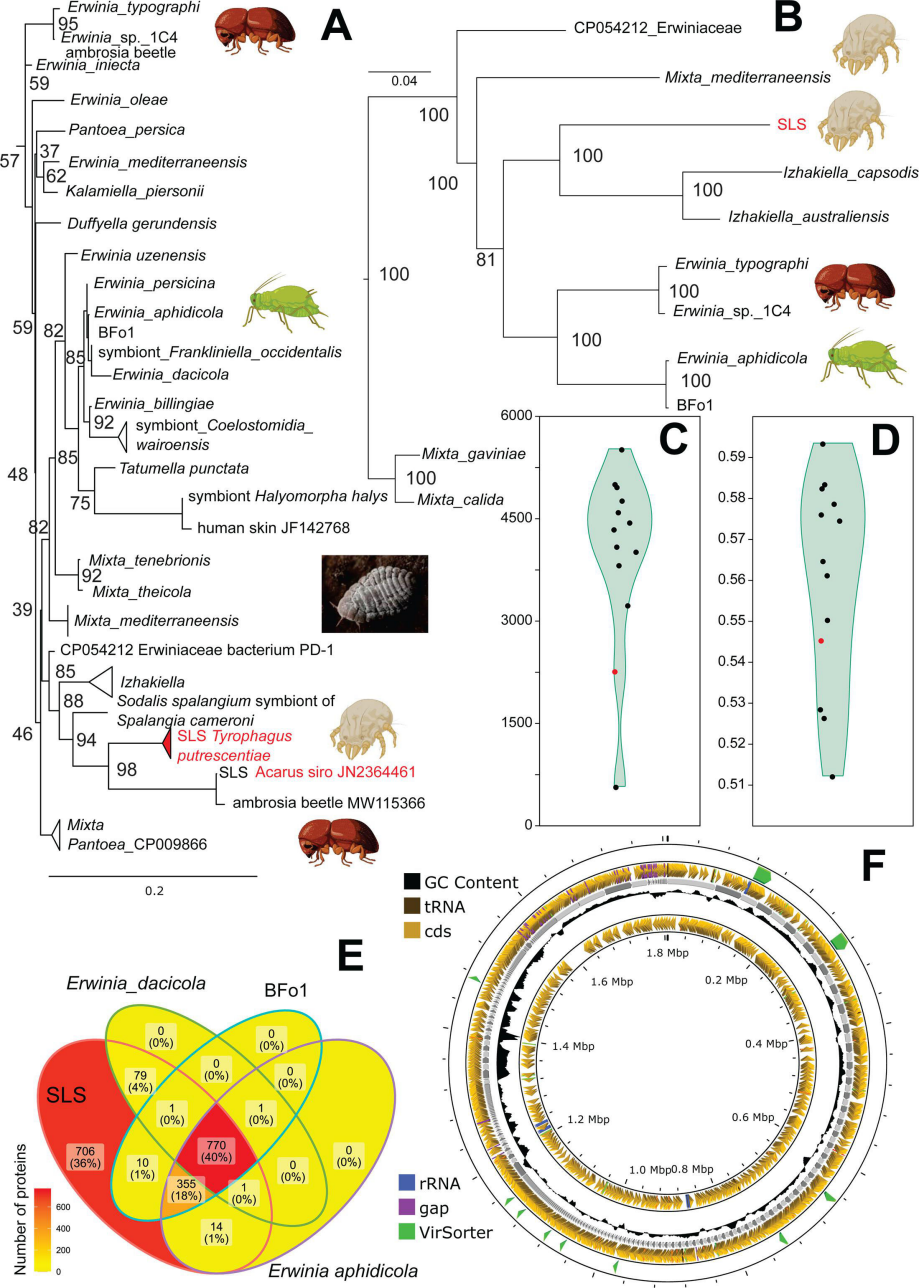
Phylogenetic affinities and genomic features of Erwiniaceae (SLS) symbiont associated with *Tyrophagus putrescentiae*: (A) 16S rDNA phylogeny with the TN93 + R111 nucleotide substitution model; (B) maximum likelihood phylogeny based on orthologous protein groups using *Mixtamixta tenebrionis* as outgroup; (C) comparison of open reading frames (ORFs), with SLS indicated by a red point; (D) GC content comparison, with SLS indicated by a red point; GenBank IDs for genomic and 16S rDNA sequences are listed in Tables S6 and S7; (E) Venn diagram showing predicted protein overlap among select bacterial species; and (F) genomic characteristics of SLS using PROKSEE; phages were identified by VirSorter.

The 16S RNA sequence of the assembly has 99% similarity with 1,492 bp Sanger sequences of “*Sodali*s-like symbiont,” which we previously described based on 16S RNA analyses (e.g., GenBank accession no. KM464189, KM464190, KM464192, KM464197, KM464198, KM464200, and KM464202) in *T. putrescentiae* and *Acarus siro* (27, 28). In addition, it also has a similarity of 99% with Illumina sequences of the V1–V3 or V4 16S fragments that we also sequenced (26, 42). The next closest hits included 94% to following taxa *Sodalis praecaptivus* (CP006569), *Sodalis spalangium* (OR464179) endosymbiont parasitoid wasp *Spalangia cameroni, Pantoea ananatis* (MN108150), and *Erwinia mediterraneensis* (NR_179668, LT996111). The phylogenic analyses of the genome and 16S RNA indicated that symbiont forms a sister clade to the genus *Izhakiella* (Fig. 2, Tables S7 through S9) in the *Erwiniaceae* family. *Erwiniaceae* symbiont from *T. putrescentiae* and *A. siro* formed one clade with 16S RNA (MW115366) of an ambrosia bark beetle (Fig. 2A) which is consistent with previous analyses (28).

The predicted proteome of SLS contains 1,540 predicted genes, of which 1,411 proteins were assigned to KEGG pathways. These proteins are involved in 13 complete modules and 215 pathways. Notably, SLS can synthesize threonine, arginine, pyridoxal phosphate, and lipoic acid. The bacterial secretion systems identified include the Sec pathway and the twin-arginine translocation (Tat) pathway. In addition, the outer membrane protein TolC has been detected. ABC transporters were found for taurine, arginine, glutamate/aspartate, cysteine, and phosphate. SLS also can convert ammonia into glutamate via glnA, gltB, and gltD.

A previous study using barcoding sequencing of V4 16S rRNA showed that SLS was present only in the 5L culture (42). This finding was confirmed here by using specific primers (SLS_R1 and SLS_R2). We detected SLS in 5 out of 30 mite individuals from the 5L culture. qPCR analysis with specific primers (Sod_F and Sod_R) revealed that each mite body contains approximately 10^3^ copies of SLS, whereas the mite feces (SPMG) contained 10^8^ copies per g of spent growth medium (Fig. S4). SLS was not detected in mites' eggs by qPCR, which is consistent with the results obtained from V4 16S rRNA sequencing.

**Fig 3:**
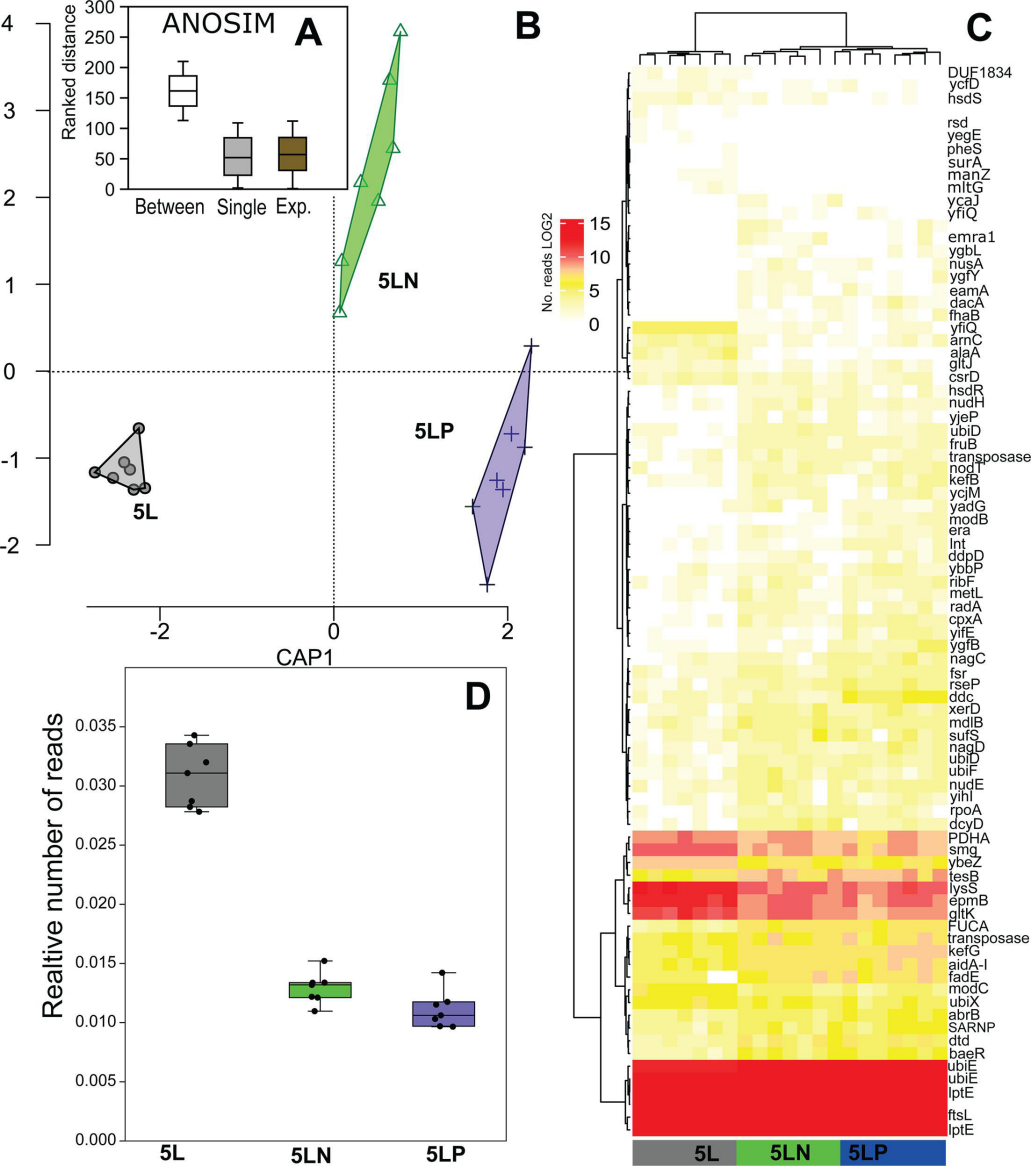
Comparison of *Erwiniaceae* symbiont (SLS) gene expression in three cultures of *Tyrophagus putrescentiae* (5L, 5LP, and 5LN): (A) ANOSIM analysis; (B) dbRDA correlation plot illustrating the distribution of samples according to SLS gene expression; (C) heatmap of SLS gene expression among the mite culturesbased on log₂-transformed values; (D) jitter and box plots showing relative numbers of SLS reads among mite cultures (SLS/mite reads), the ANOSIM comparisonshowed variability between samples (between) and inside samples.

The relative number of transcriptomic reads (SLS/mite) differed significantly among different mite cultures (Kruskal–Wallis test: *H*[chi² ] = 15.38, *P* < 0.001), with all pairwise comparisons being statistically significant (Mann–Whitney U test: *P* < 0.001). The proportion of SLS reads decreased twofold from the 5L culture to the double-infected cultures, 5LN and 5LP (Fig. 3). The expression of predicted SLS proteins (Table S10) varied among mite cultures (Table 2, model 6). The effect of mite culture type was distributed along the x-axis, explaining 14% of the total variability in the dbRDA (Fig. 3). ANOSIM indicated significant differences in SLS gene expression profiles between single- and double-infected cultures (Table S11 and S12). Using the false discovery rate (FDR), we identified 83 predicted proteins with differential expressions between single- and double-infected cultures (Table S11).

### Differences in gene expression of *Cardinium* in the samples with and without *Erwiniaceae* symbiont

**Fig 4:**
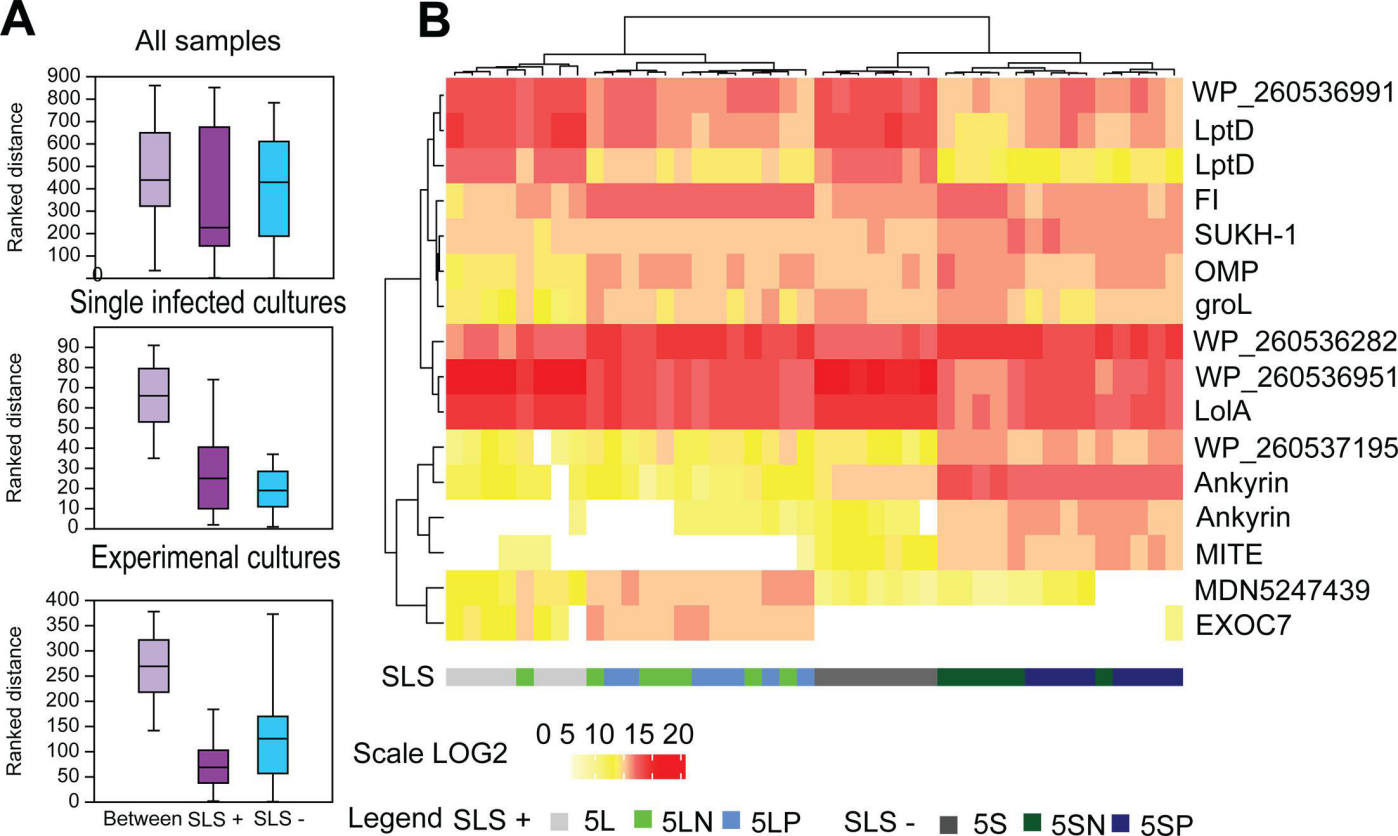
Comparison of gene expression of *Cardinium* in the samples with (SLS+) and without (SLS−) *Erwiniaceae* symbiont: (A) ANOSIM and (B) heatmap with selected genes. The ANOSIM comparison was calculated for all samples (5L, 5LP, and 5LN versus 5S, 5SN, and 5SP), single-infected cultures (*Cardinium* infection: 5L versus 5S) and experimental cultures *Cardinium*/*Wolbachia* infection: (5LN, 5LP and 5SN, 5SP), the ANOSIM comparison showed variability between samples (between) and inside samples.

**Fig 5:**
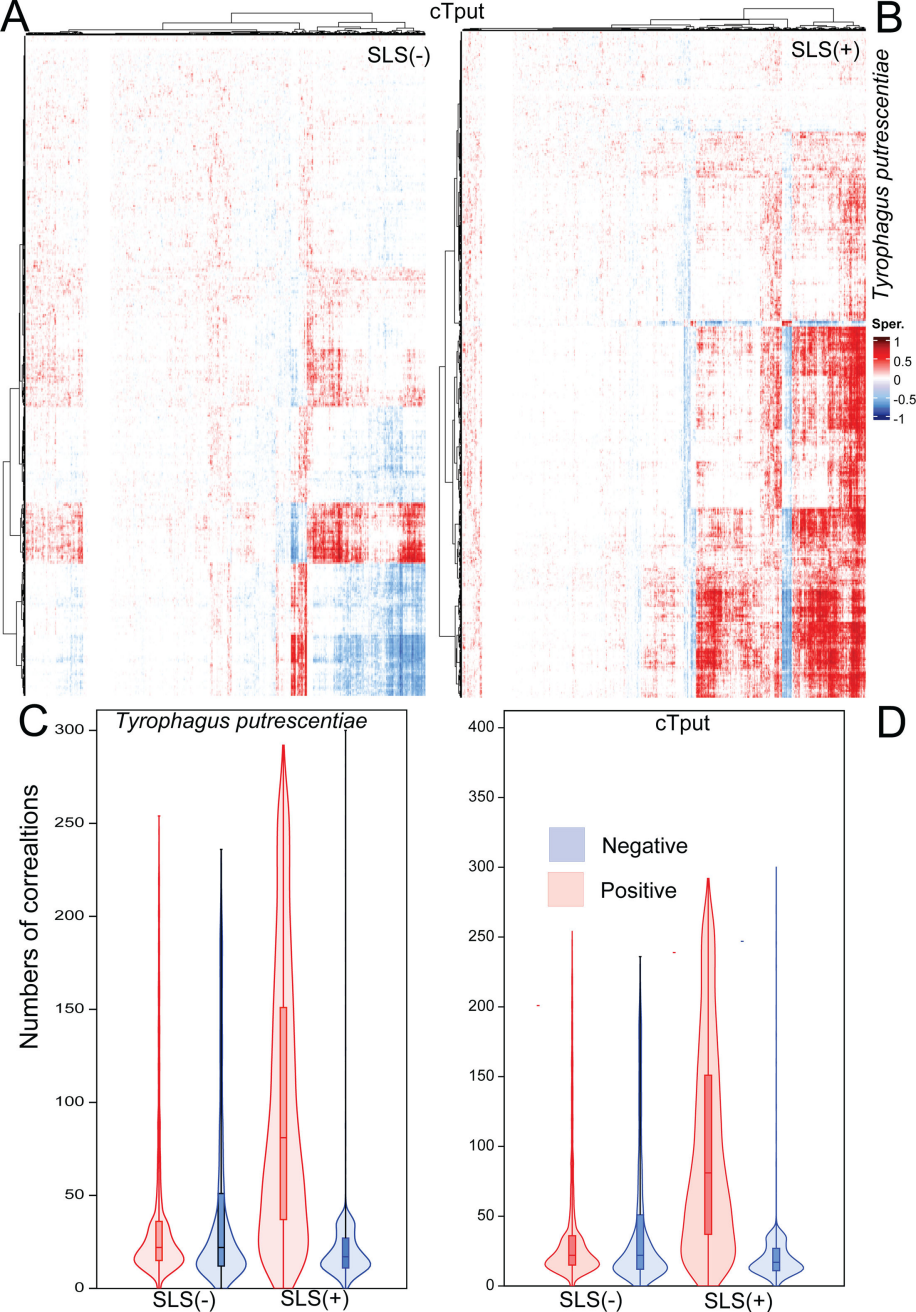
Correlational analysis between *Cardinium* (cTPut) and its mite host *Tyrophagus putrescentiae* in the samples with (SLS+) and without (SLS−) *Erwiniaceae* symbiont, based on Spearman correlation coefficients (permutational *P* < 0.05). (**A and B**) Positive and negative correlations between cTPut and the mite after Ward clustering: (**A**) SLS (−) samples, (**B**) SLS (+) samples. (**C and D**) Violin plots comparing the number of positive and negative correlations per gene: (**C**) mite vs cTPut and (**D**) cTPut vs mite.

The relative number of reads for *Cardinium* (cTPut) per mite was 100-fold lower than for SLS per mite. The cTPut reads were slightly lower in SLS-negative samples (Mann–Whitney *U* = 93, *P* = 0.001) (Fig. 4). The predicted gene expression profile of cTPut was significantly influenced by the presence of SLS (Fig. 5). However, double-infected cultures (5LP, 5LN, 5SN, 5SP) also differed from single-infected cultures (5L and 5S) (two-way ANOSIM: SLS factor *R* = 0.90945, *P* < 0.001; infection factor *R* = 1, *P* < 0.001) (Fig. 5). dbRDA analysis yielded similar results (Table 2, models 1–3), with the presence of SLS being a significant factor based on permutation tests (*P* < 0.05). cTPut gene expression was more affected by SLS gene expression than vice versa (Table 2, models 4, 5, 11, and 12). The variability in cTPut gene expression explained by SLS was 54% (Table 2, model 4), while the variability in SLS gene expression explained by cTPut was 38% (Table 2, model 11). Spearman correlation analysis revealed 39,484 positive correlations and 11,425 negative correlations between cTPut genes and SLS (Fig. S5).

FDR analyses revealed 29 upregulated and 48 downregulated cTPut genes (Tables S13 and 14) in the samples with and without SLS. Among the genes with significant expression changes and high numbers of correlations to *Erwiniaceae* symbiont, we identified six upregulated MITE genes and seven upregulated MITE-like genes, which are likely maintained by intact copies of transposase (Table S5). Of these, only the locus MDN5247515 encodes a complete IS6 family transposase protein. Other cTPut genes strongly upregulated by SLS include a hypothetical protein (MDN5247498), ParA chromosome partitioning protein (MDN5247332), centromeric protein E (CENPE; MDN5247497), and membrane-bound lytic murein transglycosylase D (mltD; MDN5247165, 99%) (Table S13).

Based on the network analyses (Table S15), the following genes exhibited a high number of interactions with *Erwiniaceae* symbiont genes among cTPut outliers: LptD proteins (WP_260537050, WP_260536905), LolA (outer membrane lipoprotein carrier protein, WP_260536850), outer membrane protein (WP_260537419), HlyD family type I (WP_260537442), IS256 family transposase (WP_260537183, complete protein), palmitoyl protein thioesterase (WP_260537224), polyribonucleotide nucleotidyl-transferase (WP_260536897), histidine-tRNA ligase (WP_26053646), ankyrin repeats (No WP), and bacteriophage tail sheath protein (WP_114910086, 99%).

### Interaction among gene expressions of the host mite and *Cardinium* and *Erwinaeceae* symbionts based on correlative evidence and Shannon diversity index

We analyzed the interaction between *Cardinium* (*cTPut*) and mite gene expression across all samples, including SLS positive (SLS+: 5L, 5LP, and 5LN) and negative samples (SLS−: 5S, 5SN, and 5SP). The predicted KEGG gene expression of the mite was significantly influenced by the presence of SLS in the samples (Table 2, model 15). The interactions between *cTPut* gene expression and predicted KEGG gene expression in the mite host were correlated (Table 2, models 16 and 18). The variance in mite KEGG gene expression explained by *cTPut* was slightly higher in the absence of SLS than in its presence (*R* = 0.476 vs 0.382; Table 2: models 16 and 18). Except for up/downregulated gene, the outliers were identified based on the comparison of the numbers of correlations to SLS or mite KEGG gene expression and position to the first axe in dbRDA models (Grubbs test *P* < 0.05) and by SIMPER analyses providing 124 cTPut genes (Table S13).

We applied the Shannon diversity index to visualize changes in gene expression across different samples. Significant differences were observed in the gene expression of both *T. putrescentiae* and *cTPut* between samples with the presence/absence of SLS (Fig. S6) (Mann–Whitney, *P* < 0.001). Gene expression diversity in the mite decreased 1.5-fold in SLS(+) samples, while *cTPut* gene expression increased by 0.1-fold in samples with SLS compared to samples without symbiont. The expression of SLS exhibited low variability (Shannon index range: 1.6–1.9) compared to *T. putrescentiae* (range: 1.1–4.2) and *cTPut* (range: 2.2–3.1), indicating that SLS expression is relatively stable. We found a significant linear relationship between the Shannon indices of *T. putrescentiae* and *cTPut* [F_(1,40)_ = 20.1; *P* < 0.001; *R*² =0.34], where mite gene expression diversity decreased as *cTPut* gene diversity increased (Fig. S5). In the samples with SLS, mite KEGG gene expression diversity decreases, while *cTPut* involves a broader range of genes in the interaction.

### Correlation networks for *Cardinium* and mite host predicted KEGG genes host in the presence or absence of *Erwiniaceae* symbiont

*Cardinium* (cTPut) predicted protein expression was influenced by mite KEGG protein expression in samples with SLS more than without SLS (Table 2, models 6 and 7). An additive effect of both mite and *Erwiniaceae* symbiont expression on cTPut expression was observed (Table 2, model 8). Spearman correlation analyses revealed 214,024 positive and 252,764 negative correlations of cTPut vs mite expression in SLS (−) cultures (Fig. S6A), whereas SLS (+) samples showed 580,306 positive and 120,158 negative correlations (Fig. S6B). These findings suggest that cTPut gene expression is modulated by both mite host genes and the presence of the *Erwiniaceae* symbiont.

To further investigate these dynamics, correlations among symbionts and mite gene expression were analyzed separately for mite immune and regulatory genes, as well as metabolic pathways. *T. putrescentiae* KEGG gene expression related to immune and regulatory pathways was influenced by both symbiotic bacteria (Table 2, models 20–23). Notably, the explained variance in mite immune regulatory pathway expression by *cTPut* gene expression increased from 38% in samples without *Erwiniaceae* symbiont to 51% in samples with *Erwiniaceae* symbiont. In addition, dbRDA models indicated an additive effect of *cTPut* and *Erwiniaceae* symbiont gene expression on the mite host, explaining 57% of the variance (Table 2, model 23). This suggests that mite immune and regulatory pathway gene expression is influenced by both *cTPut* and *Erwiniaceae* symbiont, with a different host response in *Erwiniaceae* symbiont-negative samples. Spearman correlation analyses between *cTPut* and predicted KEGG immune and regulatory pathway gene expression in the mite showed 8,582 positive and 11,714 negative correlations in samples without *Erwiniaceae* symbiont, compared to 19,071 positive and 9,823 negative correlations in samples with *Erwiniaceae* symbiont.

The Cytoscape network analyses used an absolute Spearman correlation coefficient (*P* < 0.05) from 0.75 to 1. The network was constructed from selected cTPut genes (*N* = 124) (Table S13) × mite KEGG genes involved in immune and regulatory pathways (*N* = 1,056) separately for samples with the absence (Table S16) or presence (Table S17) of SLS. The network analyses identified 21 cTPut proteins (Table 3, Fig. S7 through 12) and 54 mite KEGG proteins from immune and regulatory pathways mostly receptors of lysosome function, ubiquitin-mediated proteolysis, PIK3/Akt signaling, and cGMP-PKG pathways (e.g., cathepsins C, F, H, and O (Table 4). The identified members of the Cytoscape network from cTPut proteins showed positive correlations WP_260536282 (Fig. S13) and negative histidine-tRNA ligase (GenBank accession no. WP_26053646) (Fig. S14), or both for following proteins: ankyrin-containing proteins (Fig. S15A), hypothetical proteins (WP_260536329) (Fig. S15B), hypothetical protein 5S_8490) (Fig. S1), (MDN5247383) (Fig. S16B), (MDN5247180) (Fig. S17A), (MDN5247498) (Fig. S17B), Type IV secretion-system coupling protein DNA-binding domain (5S_8750) (Fig. S18A), transcription termination factor NusA (WP_260535886) (Fig. S18B), 60 kDa chaperonin (WP_260536873) (Fig. S19A), and signal peptide peptidase SppA (WP_260537057) (Fig. S19B). These proteins are candidates to be recognized by mite immune and regulatory pathways and influence mite response to symbionts.

**TABLE 3:** The *Cardinium* (cTPut) predicted proteins selected based on Cytoscape correlation networks comparing correlations between cTPut and mite KEGG proteins in immune and regulatory pathways expression^*a*^,^*b*^

*Cardinium* (GenBank)	ID	Name	Description	Network	cTPut × KEGG all				cTPut × KEGG immune			
					SLS (−)		SLS (+)		SLS (−)		SLS (+)	
					P	N	P	N	P	N	P	N
MDN5246949	5S_2860		Recombinase family protein^*b*^	SLS+	161	139	3,160	16	28	22	619	2
WP_114910452_96%	5S_4160		PD-(D/E)XK nuclease family transposase^*b*^	SLS−	303	1,673	2,214	16	61	332	424	2
WP_014934832_82%	5S_7760		Rpn family recombination-promoting nuclease/putative transposase^*b*^	SLS−	293	1,805	68	46	54	365	4	10
WP_144087109_81%	5S_7770		Ankyrin repeats	SLS−	473	1,324	442	22	91	253	73	3
WP_260536329	5S_5690		Ankyrin repeats	SLS+	423	859	3,444	39	100	170	724	2
WP_260536282	5S_4760		Hypothetical protein^*b*^	SLS+/−	935	395	3,637	37	208	77	731	2
UWW97528_76%	5S_8490		Hypothetical protein^*b*^	SLS−	253	1,692	43	1,081	52	339	6	208
MDN5247383	C_7540		Hypothetical protein^*b*^	SLS+	313	176	46	3,009	54	40	4	598
MDN5247180	C_5540		Inner membrane protein^*b*^	SLS+	361	92	3,186	35	51	15	637	3
MDN5247498	C_8660		Hypothetical protein^*b*^	SLS+	11	130	3,206	39	3	26	621	3
MDN5247178_94%	5S_8750		Type IV sec. coupling protein DNA-binding domain^*b*^	SLS−	1,411	365	46	1,080	296	68	4	206
WP_260536188	5S_0220	pdhA	Pyruvate dehydrogenase (acetyl-transferring) E1 comp.	SLS−	368	1488	1,068	71	68	301	183	9
WP_114910086	5S_1490	FI	Bacteriophage tail sheath protein	SLS+	111	161	2,289	52	20	33	482	5
WP_260535886	5S_1780	nusA	Transcription termination factor NusA	SLS−	335	1,539	1112	67	62	308	182	9
WP_260536645	5S_2370	accC	Acetyl-CoA carboxylase biotin carboxylase subunit	SLS+	162	32	2,962	9	29	3	669	1
WP_260537057	5S_3140	sppA	Signal peptide peptidase SppA	SLS−	213	1,634	1,675	43	39	336	335	3
WP_260536767	5S_5050		C4-type zinc ribbon domain-containing protein	SLS−	333	1,583	2,269	49	70	318	433	3
WP_260536873	5S_5270	groL	60 kDa chaperonin	SLS+	689	93	3,631	35	126	14	770	2
MCT4697279	5S_5820	PPIs	Peptidylprolyl isomerase	SLS+	1,252	213	30	3,041	249	37	3	669
MCT4696963	5S_7190	rplR	50S ribosomal protein L18	SLS+	105	94	3,730	13	14	18	749	2
WP_26053646	5S_7430	hisS	Histidine--tRNA ligase	SLS+/−	541	912	43	2,510	104	188	4	487

^*a*^ The table shows the numbers of positive (P) and negative (N) correlations between cTPut and mite KEGG-predicted proteins. The numbers included numbers from all KEGG proteins (*N* = 5,880) and selected KEGG proteins (*N* = 1,056) in immune and regulatory pathways.

^*b*^ These hypothetical proteins were compared to proteins from other *Cardinium* (WP_260536282—Fig. S6; MDN5247383—Fig. S7, MDN5247498—Fig. S8, MDN5247180—Fig. S9, 5S_8490—Fig. S10, 5S_8750 (Fig. S11)**.** The criterion for the presence of Cytoscape network was absolute values Spearman correlations coefficient in the range from 0.75 to 1 (*P* < 0.05). The networks were analyzed for the samples with (SLS+) and without (SLS−) *Erwiniaceae* symbionts.,

**TABLE 4:** Predicted mite *Tyrophagus putrescentiae* KEGG proteins in immune and regulatory pathways selected based on Cytoscape correlation networks comparing correlations between *Cardinium* (cTPut) and mite KEGG protein expression^*a*^,^*b*^

KEGG	Pathways	Name	Description	Net	All cTPut				Selected cTPput			
					SLS (−)		SLS (+)		SLS (−)		SLS (+)	
					P	N	P	N	P	N	P	N
K01052	Lysozyme	LIPA	Lysosomal acid lipase/cholesteryl ester hydrolase	SLS−	30	181	146	23	17	43	25	13
K03662		ATPeVS1	V-type H+-transporting ATPase S1 subunit	SLS+	21	84	238	40	8	22	41	20
K01189		GLA	Alpha-galactosidase	SLS−	37	195	212	36	17	44	27	22
K01192		MANBA	Beta-mannosidase	SLS−	36	191	64	17	19	44	14	6
K01195		GUSB	Beta-glucuronidase	SLS−	39	211	174	36	18	49	23	21
K01201		GBA	Glucosylceramidase	SLS+	34	190	233	38	18	44	38	20
K01204		NAGA	Alpha-N-acetylgalactosaminidase	SLS+	35	193	231	36	18	44	36	21
K01275		CTSC	Cathepsin C	SLS−	34	186	223	39	19	43	31	24
K01366		CTSH	Cathepsin H	SLS−	35	173	61	13	18	40	4	8
K01373		CTSF	Cathepsin F	SLS+	35	185	253	42	17	44	35	24
K01374		CTSO	Cathepsin O	SLS+	35	176	245	37	20	42	35	20
K06497		CD63	CD63 antigen	SLS−	33	171	167	28	18	39	28	19
K10532		HGSNAT	Heparan-alpha-glucosaminide N-acetyltransferase	SLS−	37	173	185	40	17	43	27	23
K12309		ELNR1	Beta-galactosidase	SLS−	40	203	45	14	18	46	10	4
K12311		LAMAN	Lysosomal alpha-mannosidase	SLS−	34	181	164	24	18	41	25	16
K12373		HEXA_B	Hexosaminidase	SLS−	40	192	244	42	17	43	34	24
K12382		PSAP	Saposin	SLS−	32	178	164	30	18	42	28	19
K04552	Ubiquitin	UBE2L3	Ubiquitin-conjugating enzyme E2 L3	SLS+	20	94	262	38	9	30	41	18
K10589		UBE3C	Ubiquitin-protein ligase E3 C	SLS−	35	170	115	17	19	41	25	9
K02207		UBE2R	Ubiquitin-conjugating enzyme E2 R	SLS+/−	31	172	274	40	17	39	42	22
K08343		ATG3	Ubiquitin-like-conjugating enzyme ATG3	SLS+	21	16	287	39	2	3	41	20
K06237	PIK3_Akt	COL4A	Collagen type IV alpha	SLS−	37	187	45	16	17	43	11	6
K06252		TN	Tenascin	SLS+	34	188	270	37	19	40	38	18
K07198		PRKAA	5′-AMP-activated protein kinase, catalytic alpha subunit	SLS+	15	18	265	37	0	1	39	21
K07207		TSC2	Tuberous sclerosis 2	SLS−	34	204	148	20	17	46	30	13
K10134		EI24	Etoposide-induced 2.4 mRNA	SLS+	24	134	283	40	12	33	40	22
K12230		PIK3AP1	Phosphoinositide 3-kinase adapter protein 1	SLS+	24	123	40	229	13	24	21	37
K04952	cGMP-PKG	CNGB1	Cyclic nucleotide-gated channel beta 1	SLS+	94	26	272	35	12	7	39	17
K05863		SLC25A4S	Solute carrier family 25 (mitochondrial adenine nucleotide translocator), member 4/5/6/31	SLS−	38	182	124	25	18	42	19	15
K01539		ATP1A	Sodium/potassium-transporting ATPase subunit alpha	SLS−	31	161	129	28	17	39	22	19
K01540		ATP1B	Sodium/potassium-transporting ATPase subunit beta	SLS−	38	162	193	33	19	40	28	23
K03898	Rest	KNG	Kininogen	SLS+	37	179	265	37	17	43	44	21
K04412		STK3	Serine/threonine kinase 3	SLS+	17	78	245	37	10	20	36	20
K04496		CTBP	C-terminal-binding protein	SLS-	246	33	21	13	39	15	1	4
K04505		PSEN1	Presenilin 1	SLS+	22	32	264	41	5	7	36	22
K04702		PIM1	Proto-oncogene serine/threonine-protein kinase Pim-1	SLS−	223	35	59	17	43	17	11	8
K05678		ABCD4	ATP-binding cassette, subfamily D (ALD), member 4	SLS+/−	35	184	263	44	19	44	35	25
K05700		VCL	Vinculin	SLS−	32	191	142	22	17	43	26	14
K05948		FNG	Fringe	SLS+	14	60	227	36	3	6	34	20
K06059		ADAM17	Disintegrin and metalloproteinase domain-containing protein 17	SLS+/−	38	184	277	40	17	44	38	25
K06482		ITGA3	Integrin alpha 3	SLS−	29	174	52	17	16	41	14	6
K06560		CD206	Mannose receptor, C type	SLS+	36	197	263	41	17	44	38	24
K08272		CAB39	Calcium-binding protein 39	SLS+	27	152	257	42	15	37	40	21
K10396		KIF5	Kinesin family member 5	SLS+	112	27	243	33	22	9	41	18
K10523		SPOP	Speckle-type POZ protein	SLS+	12	97	289	37	9	27	44	19
K12183		TSG101	ESCRT-I complex subunit TSG101	SLS−	33	198	113	18	19	42	19	8
K12349		ASAH2	Neutral ceramidase	SLS−	32	157	142	26	17	37	25	16
K12483		EHD1	EH domain-containing protein 1	SLS−	34	162	216	40	18	39	31	22
K12749		MYL3	Myosin light chain 3	SLS+	32	123	271	36	13	28	42	22
K17776		MTX	Metaxin	SLS+	11	47	258	38	4	12	40	20
K01084		G6PC	Glucose-6-phosphatase	SLS−	239	33	145	28	37	14	21	18
K02151		ATPeV1F	V-type H+-transporting ATPase subunit F	SLS−	35	171	23	21	17	44	4	3
K02732		PSMB1	20S proteasome subunit beta 6	SLS+	32	165	38	274	17	35	22	38
K02736		PSMB4	20S proteasome subunit beta 7	SLS+	11	37	230	35	1	8	38	18

^*a*^ The table shows the number of positive (P) and negative (N) correlations between all cTPut predicted proteins (*N* = 950) or selected cTPut proteins (outliers, *N* = 124, Table S13) and mite KEGG proteins in immune and regulatory pathways.

^*b*^ The criterion for the presence in the Cytoscape correlation network was absolute values Spearman correlations coefficient in the range from 0.75 to 1 (*P* < 0.05). The networks were analyzed for the samples with (SLS+) and without (SLS−) *Erwiniaceae* symbionts.

### Correlation networks for *Erwiniaceae* symbiont and predicted mite KEGG genes

The Cytoscape network analyses used an absolute Spearman correlation coefficient (*P* < 0.05) from 0.75 to 1. The network was constructed from selected SLS genes (*N* = 91) and mite KEGG genes involved in immune and regulatory pathways (*N* = 1,056). The selection of SLS genes was based on differences in the expression in the samples with the single *Cardinium* (5L) and multiple *Cardinium* and *Wolbachia* infection (5LN and 5LP) using FDR (*N* = 83) (Table S11). Next SLS genes (*N* = 8) were outliers (Grubbs test *P* < 0.05) in the numbers of positive/negative correlations to mite KEGG genes. The networks (Table S18) included 628 mite KEGG genes from immune and regulatory pathways and 80 SLS genes. Among mite proteins with the highest level of interaction (SUM of strength >100; Table S18) belonged signaling proteins of the PI3K-Akt signaling pathway (PIK3AP1), Lysosome (cathepsin F), proteasome (PSMB1) and Phagosome (MRC). It indicates that symbiont stimulates lysosomes for its regulation.

### The correlation-based comparison of the effect of *Cardinium* and *Erwiniaceae* symbionts gene expression on mite immune and regulatory pathways expression

The partial dbRDA models were created, and mite KEGG genes were picked up according to the presence of the gene in the immune and regulatory pathways (41 data sets; Table S19). The selected predicted gene expression was tested as a dependent variable (i.e., each pathway separately). The independent variables were constructed from the following combinations: (i) cTPut gene expression in the samples with the absence of SLS; (ii) cTPut gene expression in the samples with the presence of SLS; (iii) SLS gene expression; and (iv) both cTPut and SLS gene expression. Partial dbRDA models (Table S16) demonstrate how the symbiont gene expression explains the variability in the mite KEGG pathways according to the combination of tested factors (Table 5). Then the explained variability for every immune and regulatory pathway was clustered by k means clustering and it provided five clusters (Table 5).

**TABLE 5:** Comparison of explained variability in gene expression of mite *Tyrophagus putrescentiae* KEGG immune and regulatory pathways using different explanatory variables in partial dbRDA models using gene expression of the symbionts *Cardinium* (cTPut) and *Erwiniaceae* symbiont (SLS) as the factors^*a*^,^*b*^

Immune, signaling, and regulatory pathway	Cluster	Silhouette	cTPut_SLS−	cTPut_SLS+	SLS	cTPut/ SLS
TNF	1	0.239	0.95	0.90	0.91	0.94
NOD_like	1	0.207	0.89	0.92	0.95	0.94
TGF-beta	1	0.162	0.94	0.90	0.95	0.92
Apoptosis	1	0.158	0.94	0.93	0.91	0.95
Insulin signaling pathway	1	0.112	0.91	0.83	0.94	0.95
Phagosome	1	0.091	0.91	0.93	0.94	0.95
Apelin	1	−0.006	0.89	0.94	0.92	0.93
NF-kappa B signaling pathway	1	−0.060	0.90	0.94	0.95	0.95
Sphingolipid signaling pathway	1	−0.127	0.92	0.95	0.93	0.95
JAK-STAK	1	−0.211	0.96	0.92	0.96	0.95
Hedgehog signaling pathway	2	0.303	0.82	0.95	0.93	0.95
Mitophagy	2	0.287	0.85	0.93	0.91	0.89
AMPK signaling pathway	2	0.264	0.78	0.95	0.94	0.96
Oocyte meiosis	2	0.263	0.82	0.87	0.91	0.92
Hippo	2	0.255	0.85	0.94	0.86	0.94
ErbB	2	0.247	0.85	0.92	0.92	0.95
p53	2	0.229	0.87	0.91	0.90	0.91
Bact invasion of EC	2	0.208	0.86	0.93	0.92	0.92
Wnt	2	0.170	0.80	0.94	0.86	0.91
mTOR	2	0.160	0.79	0.87	0.93	0.90
Proteosome	2	0.158	0.87	0.90	0.90	0.91
Lysozyme	2	−0.030	0.88	0.89	0.91	0.93
cGMP-PKG signaling pathway	2	−0.082	0.88	0.93	0.90	0.95
HIF-1	3	0.330	0.94	0.97	0.98	0.95
TOLL	3	0.262	0.95	0.97	0.97	0.92
TOLL_IMD	3	0.106	0.91	0.98	0.98	0.99
Phosphatidylinositol signaling	3	0.061	0.93	0.96	0.95	0.94
Notch	3	−0.011	0.95	0.95	0.95	0.93
Ubiquitin-mediated proteolysis	4	0.137	0.76	0.83	0.81	0.91
Endocytosis	4	0.063	0.76	0.87	0.80	0.83
PI3K-Akt	4	−0.001	0.83	0.73	0.85	0.83
Peroxisome	4	−0.013	0.75	0.83	0.88	0.91
cAMP signaling pathway	4	−0.223	0.83	0.85	0.82	0.83
FoxO	4	−0.244	0.79	0.89	0.87	0.88
Calcium	4	−0.297	0.85	0.81	0.80	0.88
Rap1	5	0.279	0.88	0.90	0.80	0.89
Autophagy	5	0.157	0.90	0.90	0.86	0.82
Ras signaling pathway	5	0.119	0.86	0.88	0.77	0.89
MAK	5	0.093	0.86	0.85	0.81	0.89
Actin	5	0.032	0.88	0.88	0.88	0.87
Phospholipase D signaling pathway	5	−0.079	0.87	0.88	0.88	0.88

^*a*^ The table shows the explained variability of the models with tested variables for 41 pathways. Data were clustered using k-means clustering (WGSS = 0.1205, F = 2.477, Var = 71%, average silhouette = 0.092). Predicted clusters and silhouette values are shown.

^*b*^ The details of the models are described in Table S19. The independent variables were constructed from the following combinations: (i) *Cardinium* (cTPut) gene expression in the samples with the absence of *Erwiniaceae* symbionts (cTPut_SLS−); (ii) *Cardinium* (cTPut) gene expression in the samples with the presence of *Erwiniaceae* symbiont (cTPut_SLS+); (iii) *Erwiniaceae* symbiont gene expression in the samples with the presence of *Erwiniaceae* symbiont (SLS); and (iv) both *Cardinium* (cTPut) and *Erwinaceae* (SLS) symbionts gene expression in the samples with the presence of *Erwiniaceae* symbiont (cTput/SLS).

Cluster 1 had similar levels of explained variability (*R*) among the bacterial symbionts and samples, including the TNF pathway, apoptosis, and TGF-beta pathways (range: 0.83–0.96). Cluster 2 showed lower explained variability by cTPut in the samples without SLS (range: 0.78–0.88) compared to samples with SLS (range: 0.87–0.95), including oocyte meiosis, lysozyme, and Wnt pathways. Cluster 3 contained highly influenced pathways, such as TOLL, HIF-1, phosphatidylinositol signaling, and Notch signaling pathways (range: 0.91–0.98). Clusters 4 and 5 included pathways with low explained variability (range: 0.73–0.91). Cluster 5 showed similar levels of variability explained by cTPut in both types of samples with and without SLS. By contrast, cluster 4 exhibited differences in the variability explained by cTPut in samples without SLS (range: 0.75–0.85) than in the samples with SLS (range: 0.73–0.89).

### The effect of *Cardinium* and *Erwiniaceae* symbionts on mite metabolic pathways expression

dbRDA analyses revealed that the expression of KEGG gene profiles in metabolic pathways related to mite metabolism was significantly influenced by the presence of SLS (Table 6, model 1). In addition, double-infected cultures (5LP, 5LN, 5SN, and 5SP) differed from single-infected cultures (5L and 5S) (two-way ANOSIM: factor SLS, *R* = 0.98112, *P* < 0.001; factor infection, *R* = 0.73216, *P* < 0.001). Both glycolysis and citrate cycle expression in mites were 1.5 times lower in the presence of SLS compared to the samples without SLS (Table S20 and 23). The dbRDA models, using mite metabolism expression as the dependent variable and symbiont gene expression as the explanatory factor, showed that cTPut expression in SLS (−) samples explained 95% of the variability in metabolic pathways. By contrast, the explained variability was twofold lower in SLS (+) samples (47%) (Table 4, models 2 and 3), indicating a reduced effect of cTPut on metabolism in SLS (+) samples. Neither SLS alone nor in combination with cTPut contributed to explaining metabolism pathway expression (Table 6, models 4 and 5).

**TABLE 6:** The distance-based redundancy analyses (dbRDA) models describe the correlation between the genes involved in mite *Tyrophagus putrescentiae* metabolism expression and *Cardinium* (cTPut) and *Erwiniaceae* (SLS) symbiont gene expression^*a*^,^*b*^

No.	Dependent variable	Independent variable	Data sets	df	*F*	*R*
1	Metabolism	SLS	Presence	1	7.438	0.244
		wTPut	Presence	1	5.138	
		Residual		39		
2	Metabolism_SLS−	cTPut	Expression	9	22.701	0.949
3	Metabolism_SLS+	cTPut_SLS+	Expression	6	2.045	0.467
4	Metabolism_SLS+	SLS	Expression	4	2.561	0.390
5	Metabolism_SLS+	cTPut/SLS_SLS+	Expression	3	2.589	0.393

^*a*^ The mite metabolism pathways are based on the sum of KEGG gene expression.

^*b*^ The degrees of freedom (df), permutational test level (*F*), and explained variability (*R*) are shown. All models and parameters were significant (*P* < 0.05). The used distance was in Robust Aitchison. The samples with the presence of *Erwiniaceae* symbionts (SLS+) include 5L, 5LP, and 5LN, the samples without *Erwiniaceae* symbionts (SLS−) include 5S, 5SP, and 5SN); SLS—data set with expression of gene of *Erwiniaceae* symbiont; residuals are residual variability of the model not explained by tested variables.

## DISCUSSION

Our study aimed to investigate the variation in gene expression of *Cardinium* (cTPut), an obligate endosymbiotic intracellular bacterium associated with the stored product mite *T. putrescentiae*. We specifically tested whether (i) there are distinct cTPut genomes and (ii) the differences in cTPut expression are a response to the presence of another endosymbiotic bacterial species, such as *Erwiniaceae* symbiotic bacterium (SLS). We found that the cTPut genomes from 5L and 5S cultures are highly similar (ANI = 99%; see the following section), leading us to focus on the second hypothesis (see the sections “*Erwiniaceae* symbiont of *T. putrescentiae*” and “Interaction among cTPut, *Erwiniaceae* symbiont and the mite host”).

### Genomic similarity of *Cardinium* in two *T. putrescentiae* mite cultures

Although an ANI difference of 1% between the two cTPut genomes might be considered substantial, most of these differences are attributed to IS-derived miniature inverted-repeat transposable elements (MITEs), that is, transposase of IS256, IS4, and IS6 families (44). Here, based on HMMER identification, we propose the presence of other MITE candidates, including AAA family ATPase protein, sodium: solute symporter family proteins/HD domain-containing protein fragments (i.e., 81 elements Table S5). The AAA family ATPase fragments belong to DUF1703 family proteins consisting of an N-terminal AAA-ATPase-like domain (Pfam ID: PF09820) and a C-terminal PDDEXK_9 nuclease domain (Pfam ID: PF08011). This protein has been detected in other *Cardinium* species, such as cCpun, cSfur, and cPpe (14). In cCpun, some of the MITE elements were reported to have higher similarity to the corresponding *Rickettsia* protein, indicating possible independent acquisition (14). A similar acquisition is expected in cTPut. Sodium/substrate symporter family (SSS) proteins catalyze the uptake of a wide variety of solutes, including sugars, proline, pantothenate, and iodide, into cells (48). These proteins are present in a greater number of genes in *Amoebophilus asiaticus* (49) and *cEper* (50). The cTPut genome contains both complete SSS genes and their fragments. The analogic situation with the occurrence of functional genes and pseudogenes was in the genome of cBtQ1, cBtQ1 contains 48 functional transposase genes and 135 transpose pseudogenes (6). Transposable elements are known mediators of genome plasticity; they can disrupt genes and induce rearrangements such as inversions, duplications, and deletions (51). The suggestion is that these elements are likely responsible for the differences (Fig. S1) observed between the cTPut genomes based on the presence and absence of the genes (Jaccard index11), that is, between 5L (JAZHEU01) and 5S (JAZHET01), as well as previously assembled genomes from Czechia (JAUEML01) and China (JANAVR01). Detailed analyses of MITEs using long reads will be necessary for further investigation.

### *Sodalis*-like symbiont (SLS) of *T. putrescentiae*

Previous studies have shown that *Cardinium* associated with insect hosts can influence the host microbiome (30, 52). However, here we hypothesize that in the mite host *T. putrescentiae*, it is *Cardinium* (cTPut) gene expression patterns that could be modulated by the presence of another endosymbiotic bacterium, such as the *Erwiniaceae* symbiont (SLS). This symbiont was previously identified only through 16S rDNA sequences (26, 42) and suggested as “*Sodalis*-like” due to the similarity of 16S DNA to *Sodalis* (28). Here, we now report its entire genome for the first time (GenBank JAVLVR01). These genomic data allowed us to reveal a close relationship of *Erwiniaceae* to the genus *Izhakiella* within the family *Erwiniacea*e. *Izhakiella capsodes* was isolated from the mirid bug *Capsodes infuscatus* (53); while other *Izhakiella* species, such as *Izhakiella australiensis*, have been found in non-arthropod-associated environments, such as desert soil (54). Our 16S rDNA phylogenetic analysis (Fig. 2A) identified a monophyletic clade that includes *Erwiniaceae* symbiont from *T. putrescentiae* and another stored-product mite *A. siro* (JN2364461), and *Erwiniaceae* symbiont from an ambrosia beetle (MW115366). These findings suggest that (i) *Erwiniaceae* may exhibit some specificity to mites, as the host mite genera *A. siro* and *T. putrescentiae* are phylogenetically related belonging to one family, and (ii) ecologically, *Erwiniaceae* may prefer fungivorous hosts, given that both species of mites and ambrosia beetles feed on fungal mycelium. In contrast to the *Erwiniaceae* symbiont from *T. putrescentiae* and *A. siro*, the bacterium *Mixta mediterraneensis* (*Erwiniaceae*) from the mite *Blomia tropicalis* is not closely related to this symbiont (55). The presence of *Erwiniaceae* symbiont from *T. putrescentiae* in mite bodies and feces, but not in eggs, indicates that the *Erwiniaceae* symbiont occurs in the gut. A similar localization was found for *Bartonella*-like bacterium (family *Bartonellaceae*) in the same mite species (56).

Mutualistic interactions between *Erwiniaceae* and insects can benefit (21, 57, 58). Here, our genome analyses show that *Erwiniaceae* symbiont can synthesize threonine, arginine, pyridoxal-P, and lipoic acid. However, it needs to be confirmed whether these compounds are beneficial to the host. *Erwiniaceae* symbiont replaced *Solitalea*-like bacteria in the microbiome of individuals on a diet containing 1.25 µg·g^−1^ pirimiphos-methyl residue in a laboratory experiment involving stored product mite *A. siro* (28). This change in the SLS profile indicates that the symbiont is influenced by pesticide exposure. One possible interpretation is that symbionts may enhance the host mite’s ability to survive under pesticide stress by providing essential vitamins. Further research is needed to determine the role of SLS in the diet of host mites.

### Interaction among *Cardinium*, *Erwiniacea*e symbiont, and the mite host

The lifestyle of *Cardinium* as an endosymbiont relies on the nutrition it receives from the host. It is suggested to be vertically transmitted during oogenesis by migrating from surrounding tissues into immature oocytes (59). Consequently, *Cardinium* may influence the gut bacteriome indirectly through the host rather than directly through regulatory compounds. By contrast, SLS inhabited the gut and its potential impact on the *Cardinium* can be indirect. Here, we show that (i) *Cardinium* from *T. putrescentiae* alters predicted gene expression in samples with and without *SLS*; (ii) *Cardinium* predicted gene expression influences the mite host’s gene expression in immune and regulatory pathways as well as in metabolism; and (iii) the number of upregulated *Cardinium* genes is lower in SLS-positive samples.

### *Cardinium* predicted gene expression in *Erwiniaceae* symbiont-positive samples

The differences in genome expression of *Cardinium* in the samples with and without SLS are interpreted as a response of *Cardinium* to SLS in our study. However, 46% (*N* = 28) of upregulated cTPut genes are IS-derived miniature inverted-repeat transposable elements (MITEs) or MITE candidates, including fragments of genes coding sodium/substrate symporter (SSS) proteins and DUF1703 domain family proteins (AAA family ATPase) (Table S13). This observation aligns with previous research, which found significant expression of transposases in cEPer from *Encarsia suzannae* (50). The upregulation of MITEs in SLS-positive samples may reflect a response to symbiont competition and warrants further investigation.

Other upregulated genes are involved in peptidoglycan biosynthesis and degradation, such as *mltD* and *murl*. Glutamate racemase (*murl*) is a crucial enzyme in the bacterial cell wall biosynthesis pathway (60). *MltD* is a cytoplasmic lytic transglycosylase responsible for the nonhydrolytic cleavage of the β-1,4 glycosidic bond between N-acetylmuramic acid (MurNAc) and N-acetylglucosamine (GlcNAc), producing muropeptide products with an 1,6-anhydro-MurNAc (anhMurNAc) (61). The upregulation of these proteins suggests that *Cardinium* may be self-regulating rather than acting directly against *Erwiniaceae* symbionts. This is further supported by the upregulation of proteins associated with genetic information processing, such as *GCF*, *parA*, *CENPE*, and *rimL*. However, the number of *Cardinium* reads remains similar across samples, indicating that the observed changes do not suggest accelerated *Cardinium* multiplication in the samples with SLS.

Among the downregulated proteins in *Cardinium*, MITE or suggested MITE elements included 28% (*N* = 47) of predicted genes. Four predicted genes contained ankyrin repeat domains (ANK) and a Type IV secretion system protein, which is important for manipulating the insect host cell (51). ANK is a 33-residue motif frequently found in tandem arrays that form structures mediating protein-protein interactions essential for host cell recognition (62, 63). ANK-containing proteins are well documented in *Cardinium* and *Wolbachia* (13, 51). *Cardinium* genomes typically harbor between 18 and 54 ANKs, with approximately 50% being species-specific (13). Therefore, in the presence of SLS, the decreased levels of ANK proteins could alleviate the suppression of the host, potentially affecting the host’s physiological and immune responses.

*Cardinium* influences the mite host differently depending on the presence of SLS. We identified several hypothetical proteins (e.g., WP_260536282, MDN5247498, MDN5247383, MDN524718) that are predicted to interact with mite immune and regulatory pathways, including lysozyme, cGMP-PKG, PIK3-Akt, and ubiquitin-mediated proteolysis. Our correlation analyses revealed that in SLS-positive samples, there is a greater correlation between *Cardinium* and mite host immune and regulatory pathways compared to SLS-negative samples. In addition, *Cardinium* expression explains a higher variability in models of total host immune and regulatory KEGG gene expression in SLS-positive samples than in SLS-negative ones. This provides indirect evidence that mites interact more with *Cardinium* in the presence of the gut symbiont. The diversity of gene expressions (measured by the Shannon index) for mite hosts is greater in SLS-negative samples than in positive ones; however, cTPut reacts oppositely to mite hosts.

Mite metabolic gene expression indicates that the combination of SLS and *Cardinium* decreases the expression of genes associated with glycolysis and citrate cycles. This suppression of metabolic pathways may represent the cost of dual infection with *Cardinium* and SLS. While our correlation analyses reveal a clear impact of *Cardinium* on mite immune and regulatory pathways, distinguishing which specific mite pathways are most influenced by the *Cardinium*/SLS interaction is challenging with the current experimental design. Previous research has indicated that *Cardinium* infection induces the expression of immune-related genes in the host, including antimicrobial peptides, pattern recognition receptors, and serine proteases (64). It also affects detoxification metabolism genes, such as UDP-glucuronosyltransferase, P450 genes, and ubiquitin-related genes, as well as apoptosis-related genes (e.g., TNF receptor-associated factor 6), autophagy-related genes (e.g., gene16L), and insulin pathway genes in whiteflies (65, 66). Our study demonstrates that the presence of SLS enhances interactions between *Cardinium* and its mite host. We confirm that the expression of KEGG genes in mite immune, regulatory, and metabolic pathways correlates with *Cardinium* gene expression, as previously observed in *D. farinae* (67). In particular, cDFar gene expression explained 95% of the variation in KEGG gene expression related to various biological pathways, including phagocytosis, apoptosis, MAPK signaling, endocytosis, TNF and TGF-beta pathways, lysozyme, and the Toll/Imd pathway. Our study also confirms interactions between *Cardinium* and/or SLS with the TNF and Toll/Imd pathways and highlights significant influences on AMPK, JAK-STAT, Notch, and phosphatidylinositol signaling pathways for *Cardinium*, and HIF-1, sphingolipid signaling, and phagosome pathways for both SLS and *Cardinium*.

### Conclusion

Our study demonstrated that symbionts significantly influence host metabolism and physiology, particularly in mite immune and regulatory pathways. The effects of the intracellular symbiont *Cardinium* on its mite host vary depending on the presence or absence of gut bacterial symbionts. These findings have implications for human health, given that the model mite is a key allergen producer in human-related habitats. The interactions between *Cardinium* and gut symbionts impact mite allergen production in human environments, highlighting the importance of these symbiotic relationships in understanding and potentially managing allergen-related issues.

## MATERIALS AND METHODS

### Samples of mites and feces

*Tyrophagus putrescentiae* (Schrank, 1781) was sampled from different mite cultures (Table 7). The cultures were kept in the rearing facility of the Czech Agrifood Research Center (CARC; Crop Research Institute until 2024), Prague, Czechia. For the next comparison with *Cardinium,* we used the experimental cultures described previously (41), which included eight cultures. Mite cultivation was performed in Iwaki flasks and SPMd diet, that is, a mixture of oat flakes, wheat germs, and Mauripan (dried yeast: AB Mauri, Balakong, Malaysia) (ratio, 10:10:1 [wt/wt/wt]). The culture is renewed monthly by transferring adult mites into the new diets (17). For experiments, 42-day-old cultures were used. The samples included adults/juveniles, feces fraction (SPGM), and eggs according to the extraction procedure described previously (42). The feces fraction is the debris of diets from rearing medium after cultivation of mites, the mites are removed, but the medium still contains the fragments of their bodies. The feces are predominant, and the fraction is used for feces characterization (68, 69). The eggs and mite samples were surface-cleaned. Surface sterilization was performed on ice. The mite surfaces were cleaned by placing them in 100% ethanol, followed by vortexing for 5 s and centrifugation at 13,000 × *g* for 1 min. The supernatant was replaced with a 1:10 bleach (SAVO original [UNILEVER, Prague, Czechia]) containing 5% sodium hypochlorite) and the samples were then mixed by vortexing for 5 s and centrifuged at 13,000 × *g* for 2 min. The bleach was replaced by ddH_2_O, and this step was repeated twice to remove residual bleach. The individual mite samples included adult mites collected by the brush and stored in ethanol prior to DNA extraction. We collected one pooled sample of DNA for genome analyses, seven samples of mite bodies per culture for transcriptomic analyses as was described previously (41), six samples of mite bodies, eggs, and feces for qPCR, and 30 individuals for PCR from culture 5L (Table 7).

**TABLE 7:** The origin of *Tyrophagus putrescentiae* cultures used in the experiments and the presence of intracellular symbionts: *Cardinium* (cTPut), *Wolbachia* (wTPut), and *Erwiniaceae* symbiont (SLS)^*a*^

Mite culture	Name	Origin	Collector	Date	Diet	Intracellular symbionts		SLS			
								Genome	Transcriptome	PCR	qPCR
5L	Laboratory	Grain store, Bustehrad, Czechia	Zdarkova	1996	SPMd	cTPut		+	+	+	+
5N	Nestle	Food store, USA	Hubert	2007	F		wTPut				
5P	Phillips	Laboratory, USA	W. Phillips	2014	F		wTPut				
5S	Dried ham	Food factory, Cesena, Italy	Sala	2013	SPMd	cTPut			+		
5LN		Mix of 5L and 5P	Nesvorna	2022	SPMd	cTPut	wTPut		+		
5LP		Mix of 5S and 5P	Nesvorna	2022	SPMd	cTPut	wTPut		+		
5SN		Mix of 5S and 5N	Nesvorna	2022	SPMd	cTPut	wTPut		+		
5SP		Mix of 5S and 5N	Nesvorna	2022	SPMd	cTPut	wTPut		+		

^*a*^ A detailed list of samples and GenBank accession numbers is provided in Table S1; symbiont presence according to Hubert et al. (42). SPMd, grain derived diet; F, dog kernel diet.

### Sample processing

Single-mite PCR and qPCR were performed using a previously established protocol (17, 42). The preparation of transcriptomic and genome samples was described previously (70). Samples were then transported to the MrDNA laboratory (https://www.mrdnalab.com) for Illumina HiSeq 2000 sequencing. Illumina reads (250 bp) was deposited in GenBank (Table S1).

Read processing, genome and transcriptome assembly, and annotation were described previously (70). Illumina reads were trimmed with Trim Galore v. 0.6.10 (71) and processed using fastQC v. 0.12.0 (72). The reads were aligned in meta SPADES mode in hybrid SPADES v 3.14 (73, 74). Then contigs were processed by Prokka v. 1.14.5 (75) in metagenome mode. The predicted proteins were identified by KEGG using GhostKOALA v. 3.0 (76) into bacterial groups. The first identification indicated Gammaproteobacteria symbionts. Therefore, the reads were mapped onto reference data sets using Bowtie2 v. 2.5.4 (77, 78) and Minimap2 v. 2.28 (79) for long reads. The bacterial reference data set contained selected genomes of Gammaproteobacteria (Table S2). Mapped Illumina reads were aligned together with the PacBio reads in hybrid SPADES v 3.14 (73, 74). Bacterial sequences were annotated by Prokka v. 1.14.5 (75) using DFAST v. 1.6.0 (80) on a web server and predicted proteins were identified by KEGG using GhostKOALA v. 3.0 (76). The assembled genome was polished using Pilon v. 1.24 (81). Predicted proteins were assigned to KEGG categories, and metabolic pathways were identified using the KEGG mapper (82). Additional analysis was performed using the EggNOG Mapper v. 2 (83). Gene expression analyses of the novel bacterial symbiont were performed in CLC Workbench v. 22 (Qiagen, Venlo, Netherlands). The genome was visualized in Proksee (84).

Transcriptome expression analyses were performed using CLC Workbench v. 22 (Qiagen, Venlo, Netherlands) following the recommended protocol (85). Total read counts were used as the expression parameter. The total number of reads per sample ranged from 1.4 to 2.7 × 10^5^ reads/sample. We standardized the data to the sample 1.5 × 10^5^ reads.

### PCR and qPCR detection of *Sodalis*-like symbiont in 5L mite culture

Single-individual PCRs were conducted on mites from the 5L culture following a previously described protocol (17) using SLS-*s*pecific primers SLS_F (5′-GGGTTGTAAAGTACTTTCAGTCGT-3′) and SLS_R2 (5′-GTAGCCCTRCTCGTAAGGGCC-3′) amplifying an 804 bp fragment of the 16S rRNA gene. Each PCR contained 12.5 µL of EmeraldAmp MAX HS PCR 2× Master Mix (catalog number RR330A, Takara, Kyoto, Japan), 8.5 µL of dH₂O, 0.4 µM of each primer, and 2 µL of mite lysate, making a total reaction volume of 25 µL. Every PCR run contained positive control (genomic DNA) and ddH₂O (negative control). Amplification conditions were as follows: initial denaturation for 180 s at 95°C, followed by 35 cycles of denaturation for 30 s at 95°C, annealing for 30 s at 52°C, and elongation for 30 s at 72°C, with a final extension step of 5 min at 72°C. Using the same set of samples, we also amplified a 1,500 bp fragment of the 16S rRNA gene using universal bacterial primers (27, 86). A sample was classified as SLS positive if the SLS-specific primers successfully amplified the expected 16S rRNA fragment. The SLS-negative sample was identified when the use of SLS-specific primers resulted in no amplification, while the universal primers produced the expected product. Quantification of absolute gene abundances was performed according to a previously described protocol (42, 87, 88) using the following bacterium-specific primers: Sod_F 5′-TTTCAGTCGTGGAGGAAGGC-3′ and Sod_R 5′-CTCGCACCCTCCGTATTACC-3′, which amplify a 113 bp fragment of the 16S rRNA. The standards were prepared from the SLS_R1 and SLS_R2 PCR products following a previously described protocol (87). Amplification was performed with the following conditions: hot start activation for 1 cycle at 95°C for 60 s, followed by 40 cycles of denaturation at 95°C for 15 s, annealing at 60°C for 30 s, and a melt curve consisting of 95°C for 15 s, 60°C for 30 s, and 95°C for 15 s. All reactions were conducted in technical duplicates for six biological replicates per culture, including mite bodies, fecal fractions, and egg samples. Microbial gene abundance was normalized to a per mite, per egg, or per gram of SPGM (the debris from food and feces after mite culturing). Prior to analyses, gene abundance data were log(10)-transformed, and abundance values lower than one copy per unit (i.e., per mite or egg) were replaced with zeros.

### Molecular classification and phylogenomics

Whole-genomic taxonomic classification analyses were done on 21 May 2023 using the MASH algorithm (45) in Type (Strain) Genome Server (TYGS) at https://tygs.dsmz.de (46, 47) integrated with the List of Prokaryotic names with Standing in Nomenclature (LPSN; https://lpsn.dsmz.de) (47). 16S rRNA classification on a subset of closely related species (Table S4). If necessary, 16S rRNA gene sequences were extracted from whole genomes (Table S5) using Barrnap (89) in Galaxy (90). A set of select genomes (Table S5) was compared in M1CR0B1AL1Z3R (91) by detecting open reading frames (ORFs), finding orthologous groups, aligning orthologous sequences (92, 93), and inferring a Maximum Likelihood phylogenetic tree using RAxML with 100 bootstrap replicates (94). All trees were rooted and visualized in FigTree (95). Proteins were aligned in T-Coffee (96) and their phylogenetic trees were inferred using PHYML (97) and visualized in iTOL v.6 (98). In addition, proteins were compared in HMMER (99–101) on the Galaxy server (90).

### Comparison of cTPut genomes

To compare *Cardinium* genomes (Table 7), we created a database from predicted proteins of all cTPut assemblies. These proteins were then blasted against the protein database using PHMMER v. 3.1b1 (102) on the Galaxy server (103) and the hits were recorded using the --tblout argument. Only hits with the highest e-values were used (Table S3). Proteins exhibiting differences among the genomes were manually BLASTed against the NCBI database. To identify protein domains and assess protein completeness, we employed HMMER on the EMBL-EBI database (104). Sequences without any hits in the NCBI database were excluded from downstream analyses. We identified miniature inverted-repeat transposable elements (MITEs), likely being actively maintained by intact copies of their parental transposons (44). This identification was based on a comparison of transposase fragments in the EMBL-EBI database, under the assumption that the fragmented transposases originate from MITEs.

### Statistical analyses

Overlaps between KEGG proteins among the assemblies were visualized using Venn diagrams in the ggVennDiagram package (105) within R version 4.3.1 (106). Gene expression analyses were conducted in R using fuzzySim to determine the false discovery rates (107, 108) and the vegan package (109). Gene expression patterns were visualized using heatmaps, with Ward distances employed for clustering, in the Complex Heatmap package (110, 111). The variable “mite culture” was tested as an environmental variable in dbRDA analyses based on *Erwiniaceae* symbiont gene expression, using robust Aitchinson distances. In the next step, dbRDA models were calculated using the SLS gene expression data set as the dependent variable, with *Cardinium* or mite KEGG-assigned gene expression as the factors. The models were processed using both-ways selection with the ordistep function. The final step involved a similar analysis, but the dependent variables were replaced with independent variables. Spearman correlations among the gene expression data sets were calculated in PAST 4 (112), which was also used for visualizing the correlation network. Only correlations with a permutational *P*-value ≤ 0.05 were used. Correlation heatmaps were prepared in R using the Complex-Heatmap package (110, 111). Parametric (ANOVA) and nonparametric (Kruskal–Wallis) tests were performed using PAST. The correlation table was converted to a three-column format, and the subsequent visualization of correlation analyses was performed using Cytoscape 3.10.2 (113) with the MetScape module (114). The correlation networks were constructed at correlation levels of absolute numbers between 0.75 and 1, using only significant correlations (permutational *P* < 0.05). The networks were finalized using the compound spring embedder (CoSE) algorithm.

## Data Availability

All supporting data have been provided within the article or through supplemental data files. Sequencing data used in this study were generated and previously published (41) and were deposited in GenBank (PRJNA493156, PRJNA690683, PRJNA656450, PRJNA990474, and PRJNA706095). Our newly assembled genomes were deposited in GenBank (JAZHEU01, JAZHET01, and JAVLVR01).

## SUPPLEMENTAL MATERIAL

Supplemental figures msphere.00879-24-s0001.docx Figures S1-S19.

Supplemental tables msphere.00879-24-s0002.xlsx Tables S1-S21.

ASM does not own the copyrights to Supplemental Material that may be linked to, or accessed through, an article. The authors have granted ASM a non-exclusive, world-wide license to publish the Supplemental Material files. Please contact the corresponding author directly for reuse.
