# Supplementary material for: A novel Erwiniaceae gut symbiont modulates gene expression of the intracellular bacterium Cardinium in the stored product mite Tyrophagus putrescentiae
Source: mSphere. 2025 Mar 24;10(4):e00879-24. doi: 10.1128/msphere.00879-24 (PMC12039267; doi:10.1128/msphere.00879-24)
Supplement: Supplemental figures — Figures S1-S19. [file msphere.00879-24-s0001.docx]

**Supplementary figures S1–S19**

**Title:** A novel *Erwiniaceae* gut symbiont modulates gene expression of the intracellular bacterium *Cardinium* in the stored product mite *Tyrophagus putrescentiae*

**Authors:** J. Hubert, E. Glowska, S. E. Dowd, P. B. Klimov

**Journal:** mSphere

**
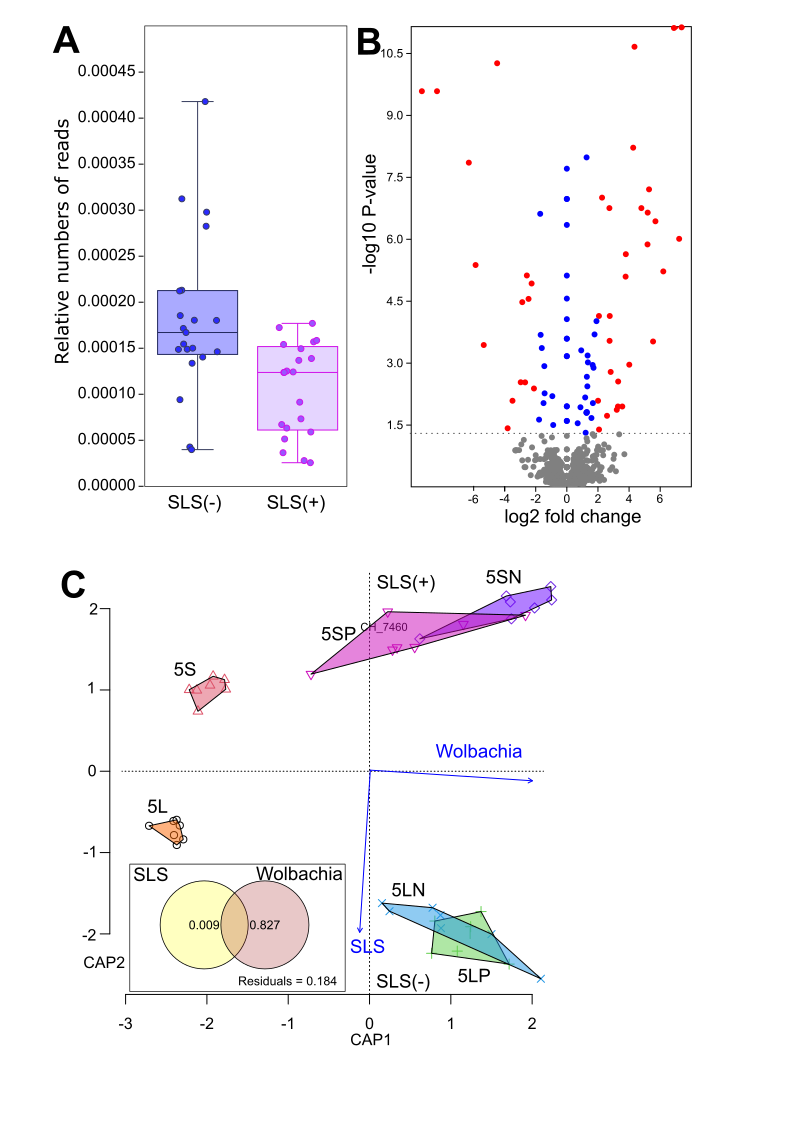
FIG S1** Gene expression of *Cardinium* (cTPut) from *Tyrophagus putrescentiae* in the samples with (SLS+) and without (SLS−) *Erwiniaceae* symbiont of *Tyrophagus putrescentiae*; **A** – Comparison of relative read numbers of *Cardinium*/mite visualized as jitter and box-whisker plots; **B** – Volcano plot illustrating the differential gene expression of *Cardinium* between SLS− and SLS+ samples as the factor. Gene expression values were LOG2-transformed. The fray color indicates samples with no significant differences (P<0.05), blue color indicates genes with significant differences in the samples, but absolute LOG2 fold change was lover than 2; red color indicates gene with significant difference and absolute LOG2 fold change higher lover than 2; **C** – The results of dbRDA analyses of *Cardinium* predicted gene expression, the tested variables were presence of *Wolbachia* and *Erwiniaceae* symbionts (SLS) showed as the vectors; samples are visualized as convex hulls; the insert is Venn diagram of explained variability. The analyzes was calculated in Robust Aitchison distance.

.


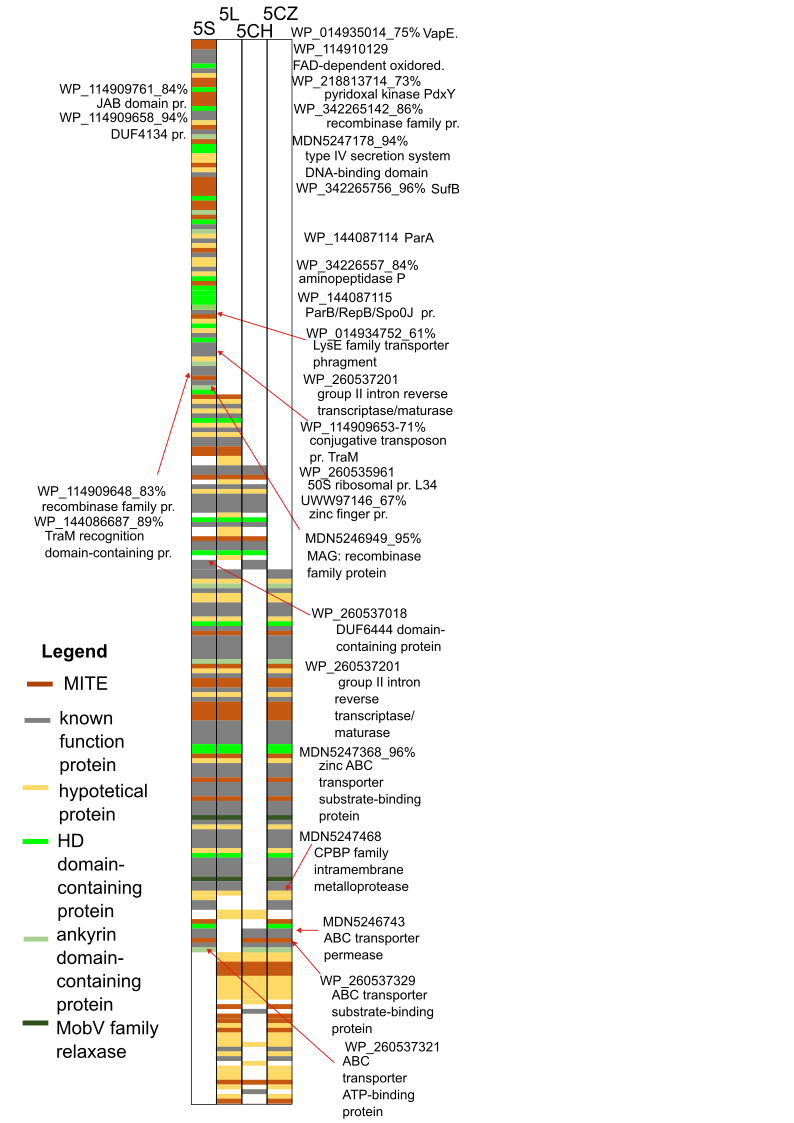
**FIG S2** Comparison of various assemblage of *Cardinium* (cTPut) symbiont of *Tyrophagus putrescentiae* predicted proteins based on SIMPER analysis, displaying the order of predicted unique proteins. The analysis compared all available proteins using PHMMER (101), with unique proteins selected based on the absence of at least one protein from four sets of predicted proteins. The following genomes were included in the comparison cTPut: 5CH (JANAVR01) from China, 5CZ (JAUEML01), 5L (JAZHEU01), and 5S (JAZHET01) from Czechia.

**FIG S3** MASH clustering of assemblage genome of *Erwiniaceae* symbiont of *Tyrophagus putrescentiae* (SLS) with the select members of *Erwiniaceae* using dREP (43) clustered on Galaxy server (103).


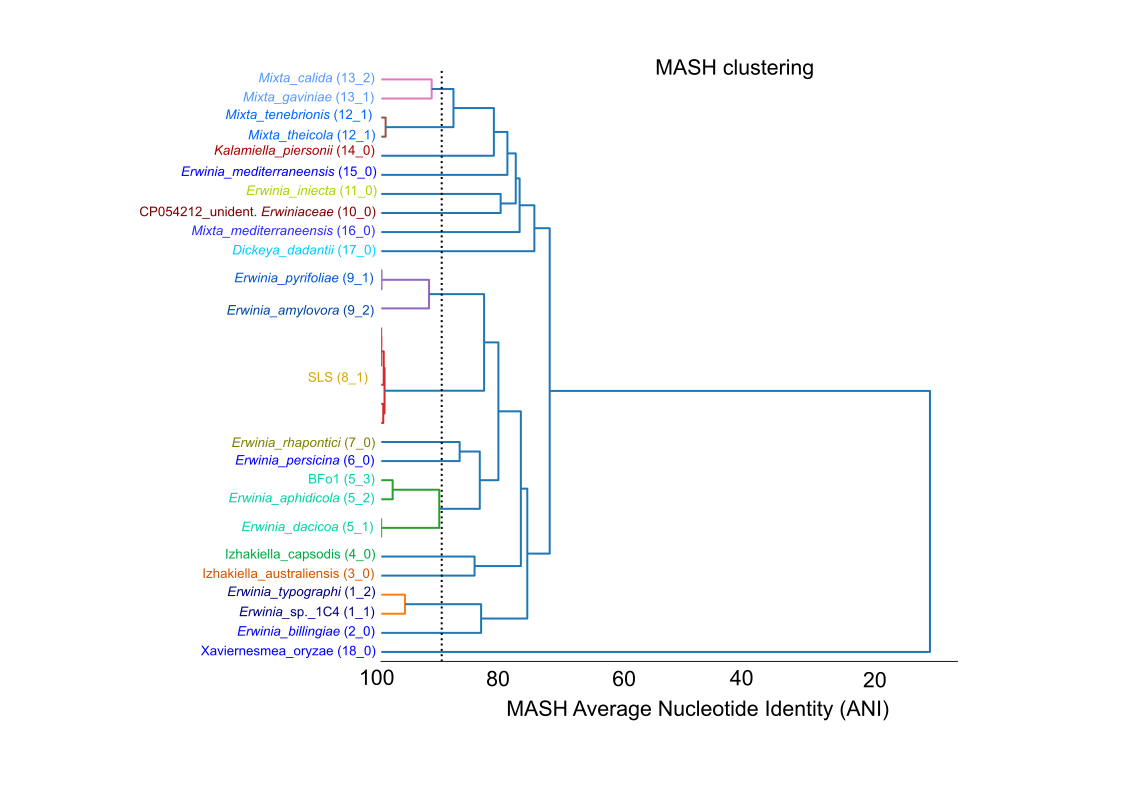


**
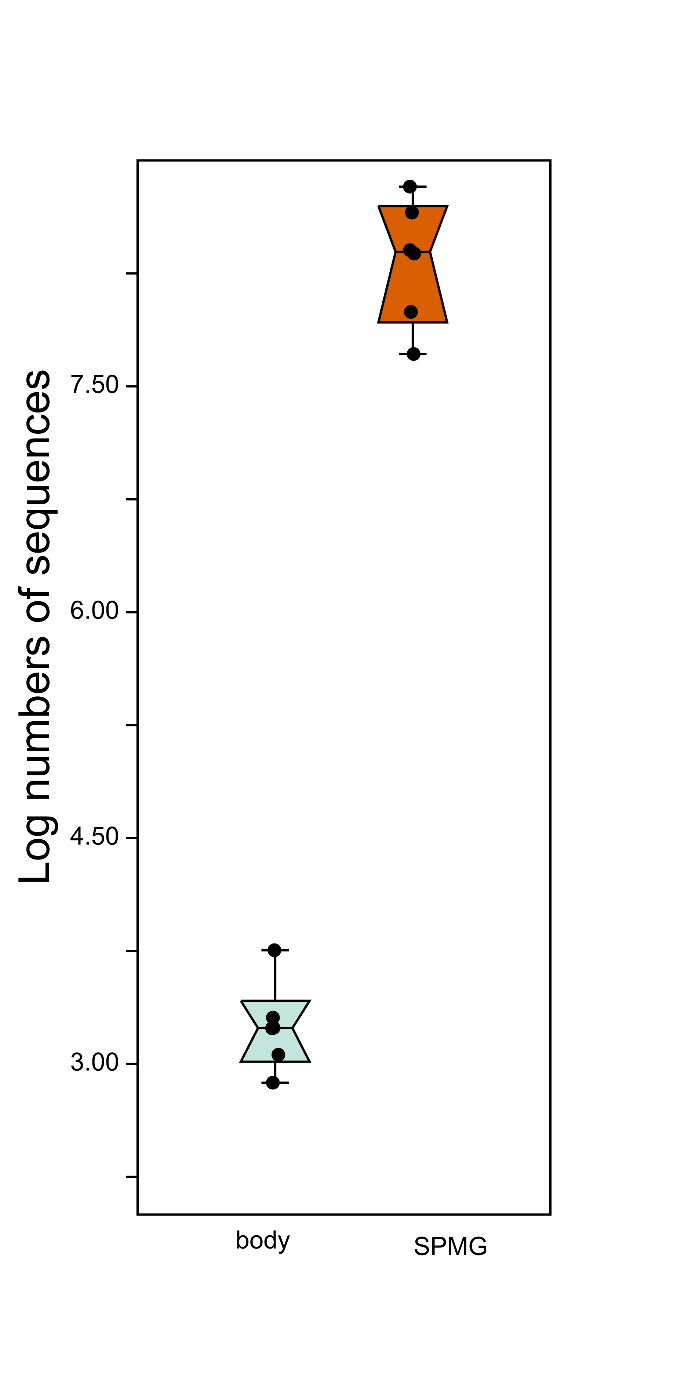
FIG S4** The numbers of sequences of *Erwiniaceae* symbionts (SLS) 16S RNA copies based on qPCR using specific primers from the sample so f mite bodies and spent growth medium (SPGM) as feces fraction of 5L *Tyrophagus putrescentiae* culture. The data were visualized as box and jitter plot. The mite bodies were recalculated as numbers of sequences per mite, while for SPGM the recalculation is per g of spent growth medium (SPGM) in the chamber. The data were LOG 10 transformed.

**FIG S5** Correlation heatmap based on Spearman correlation (permutations P < 0.05 are shown); **A** – Correlations between *Erwiniaceae* (SLS) and the mite host Tyrophagus putrescentiae; **B** – Correlations between *Cardinium* (cTPut) and *Erwiniaceae* (SLS); pairwise positive and negative correlations of predicted proteins expression are shown as violin plots: **C** – T. putrescentiae vs. SLS; **D** – SLS vs. T. putrescentiae; **E** – SLS vs. cTPut; **F** – cTPut vs. SLS.


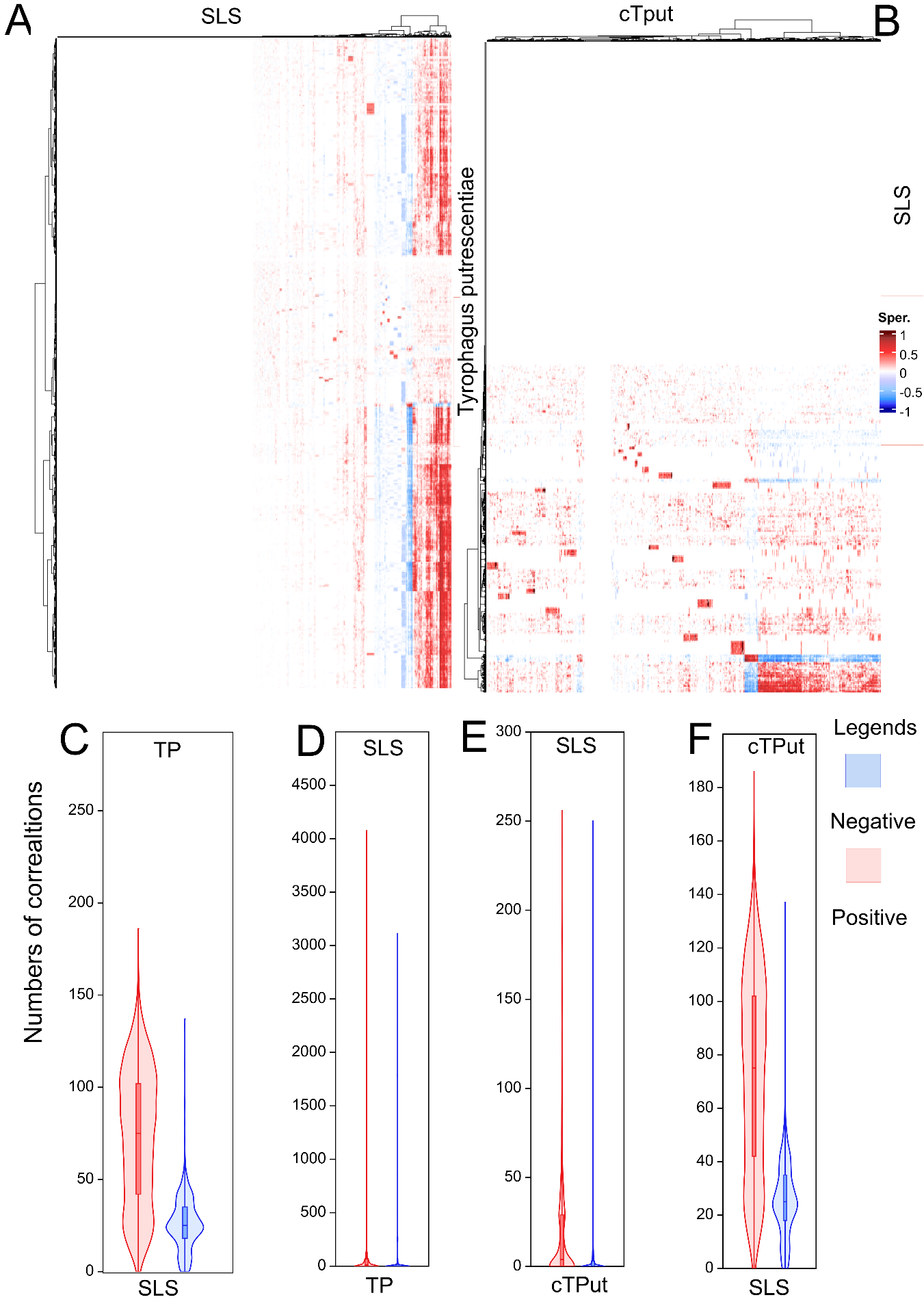


cTPut

SLS

**FIG S6** The comparison of gene expression of mite host *Tyrophagus putrescentiae*, *Cardinium* (cTPut) and *Erwiniaceae* (SLS) symbionts among the samples using Shannon diversity index. **A** – The diversity index showed negative linear response between cTPut and predicted mite KEGG gene expression. Linear regression is shown and confidence interval for regression are marked as dark red field, light red field indicates the confidence interval for model prediction. **BCD** – The diversity was compared using box and jitter plots for mite gene expression (**B**), cTput gene expression (**C**) and SLS gene expression (**D**).

**
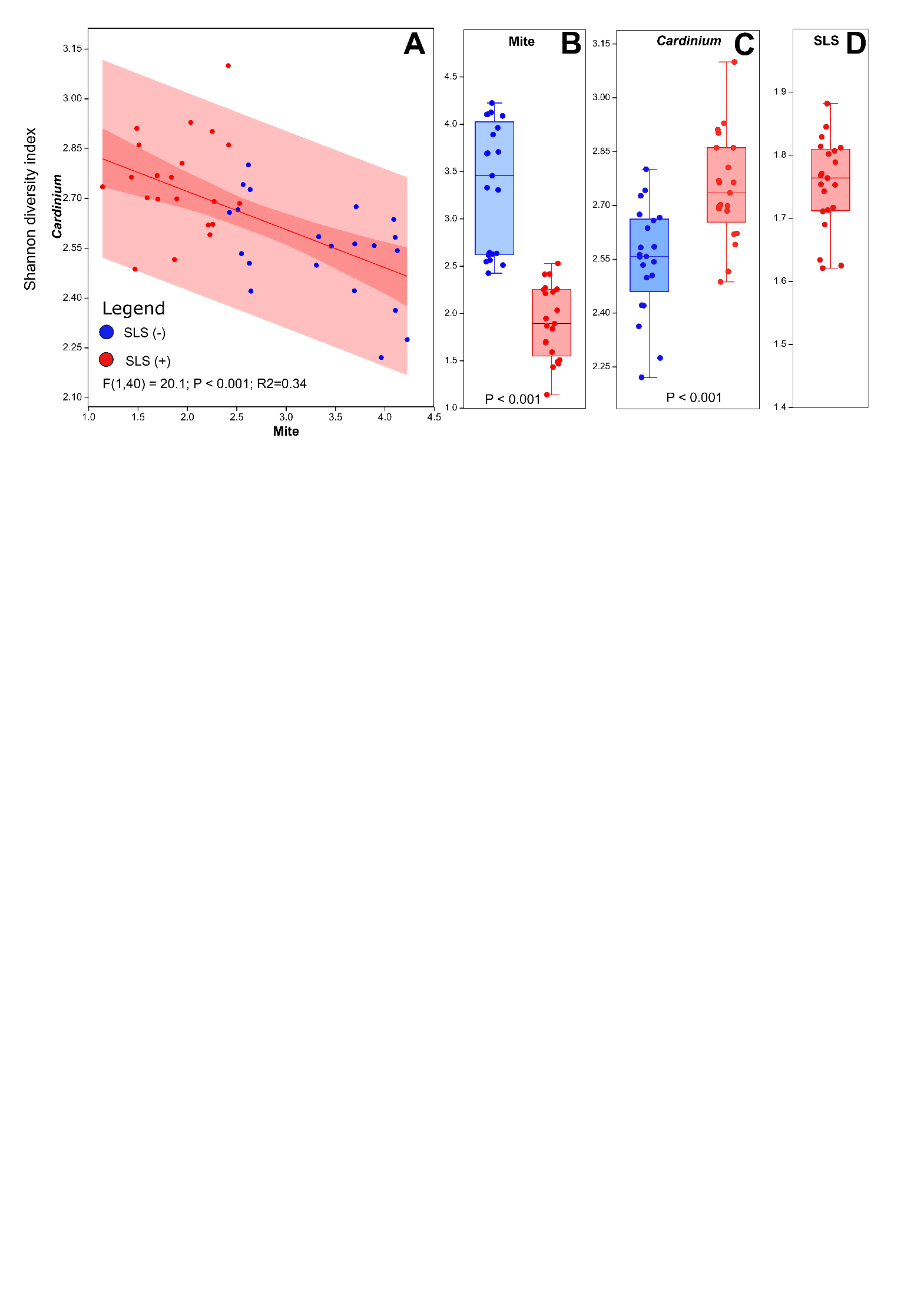
**


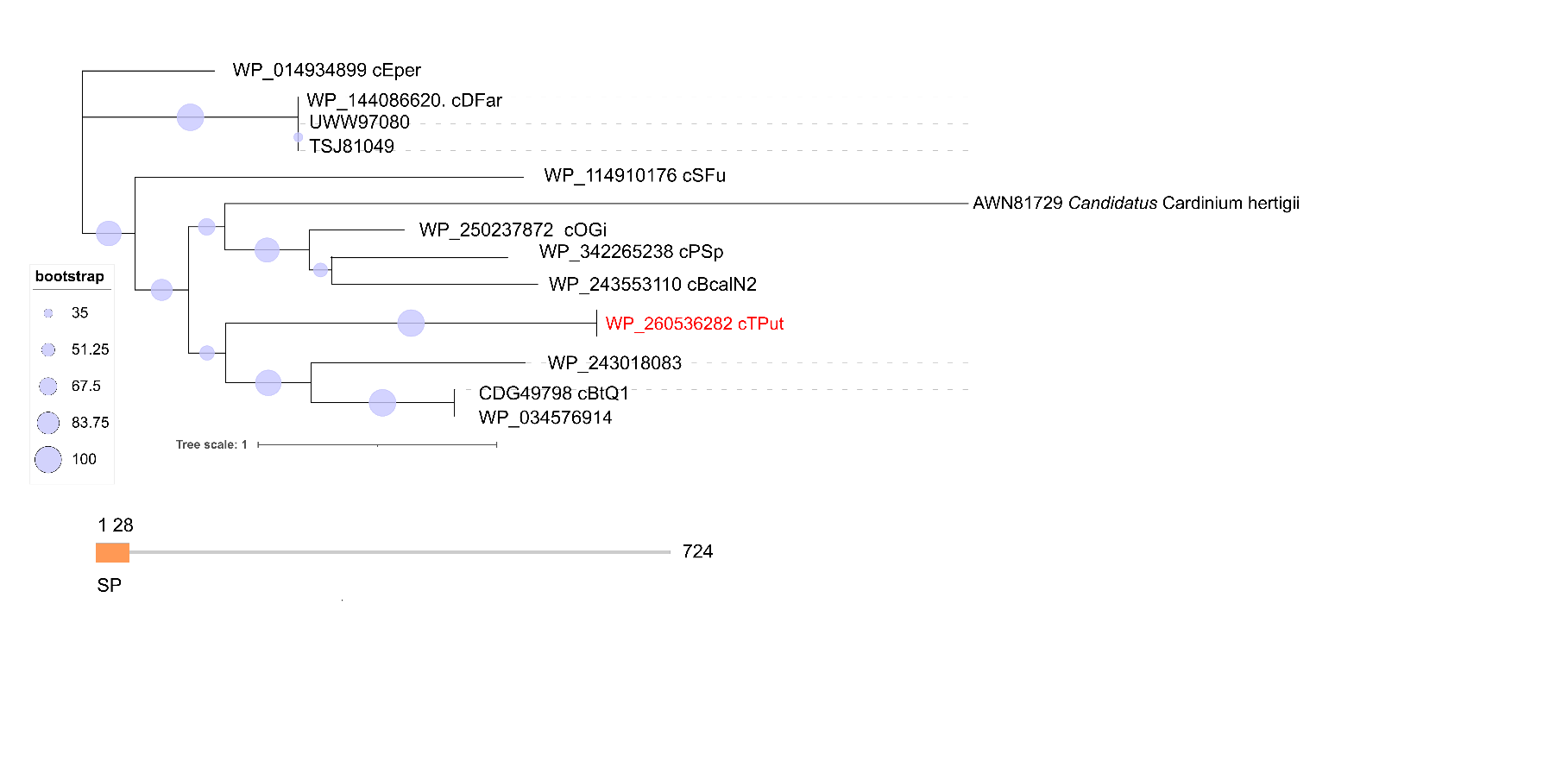
**FIG S7** Identification of the *Cardinium* 724-amino acid hypothetical protein (GenBank accession WP_260536282, red color) using GenBank data. Both unrooted tree and HMMER (101) model are shown.

Note: SP - signal peptide

**
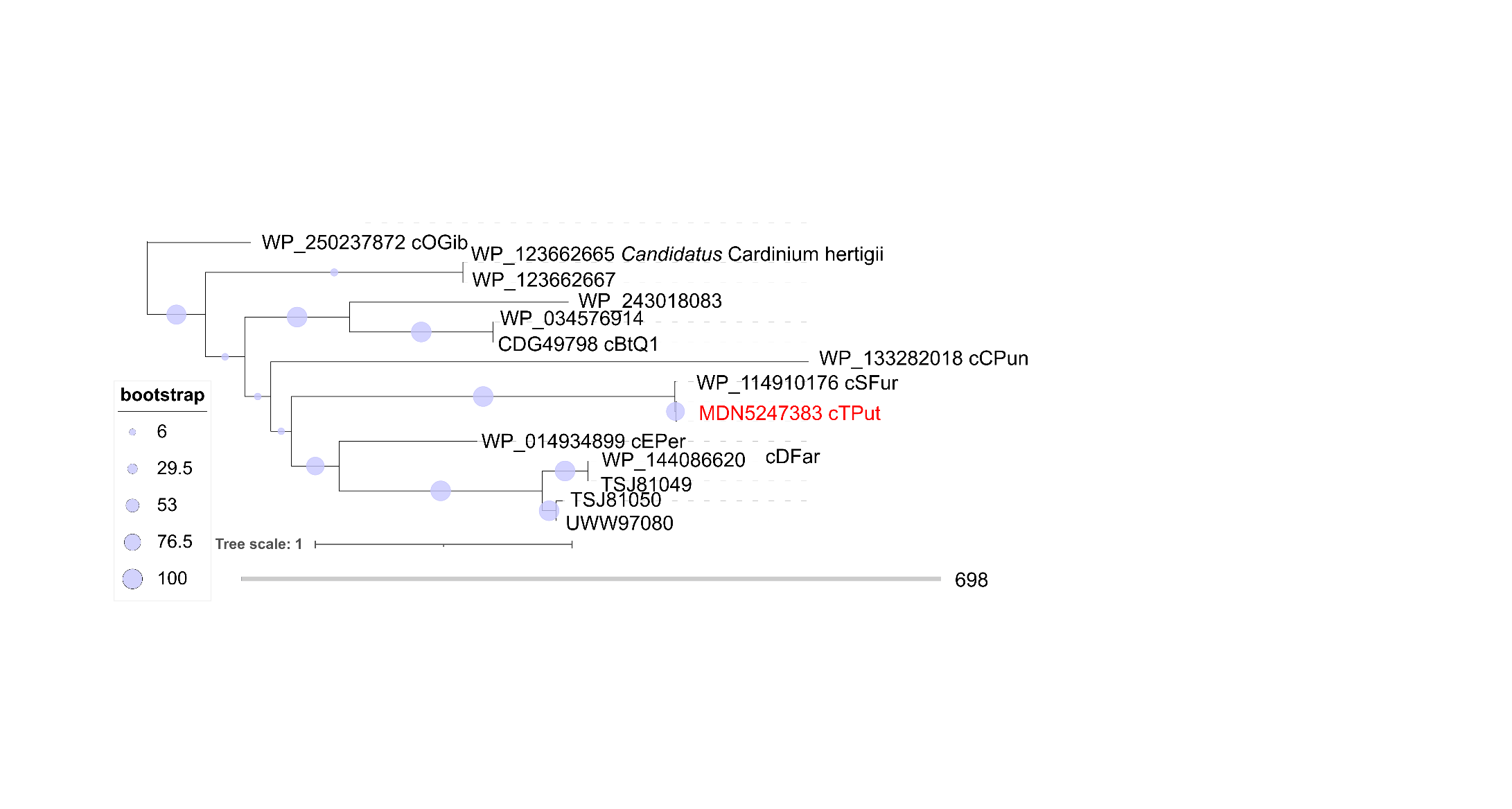
FIG S8** Identification of the *Cardinium* 698-amino acid hypothetical protein (GenBank accession MDN5247383, red color) using GenBank data. Both unrooted tree and HMMER (101) model are shown.


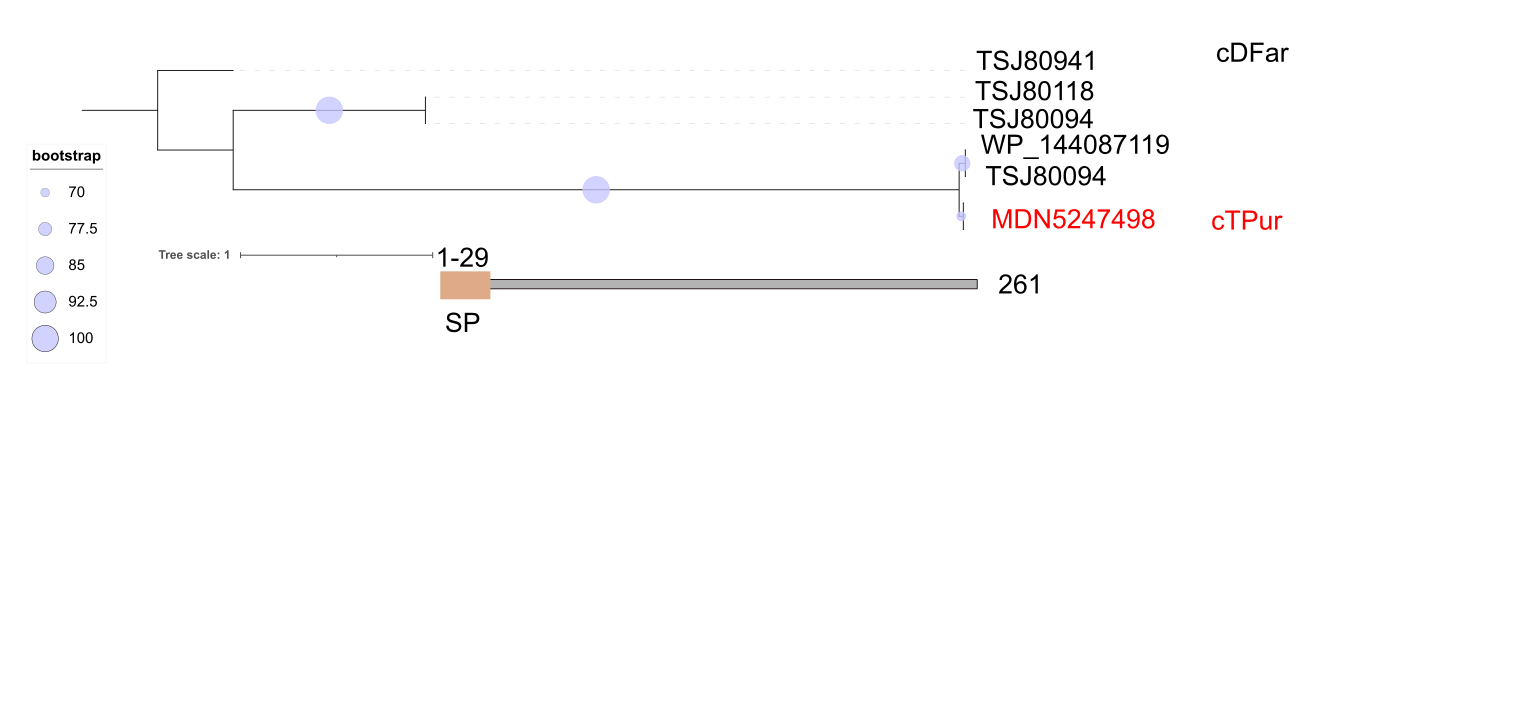
**FIG S9** Identification of the *Cardinium* 261-amino acid hypothetical protein (GenBank accession MDN5247498, red color) using GenBank data. Both unrooted tree and HMMER (101) model are shown.

Note: SP - signal peptide

**FIG S10** Identification of the *Cardinium* 193-amino acid hypothetical protein (GenBank accession MDN5247180, red color) using GenBank data. The unrooted tree and HMMER (101) model are shown.


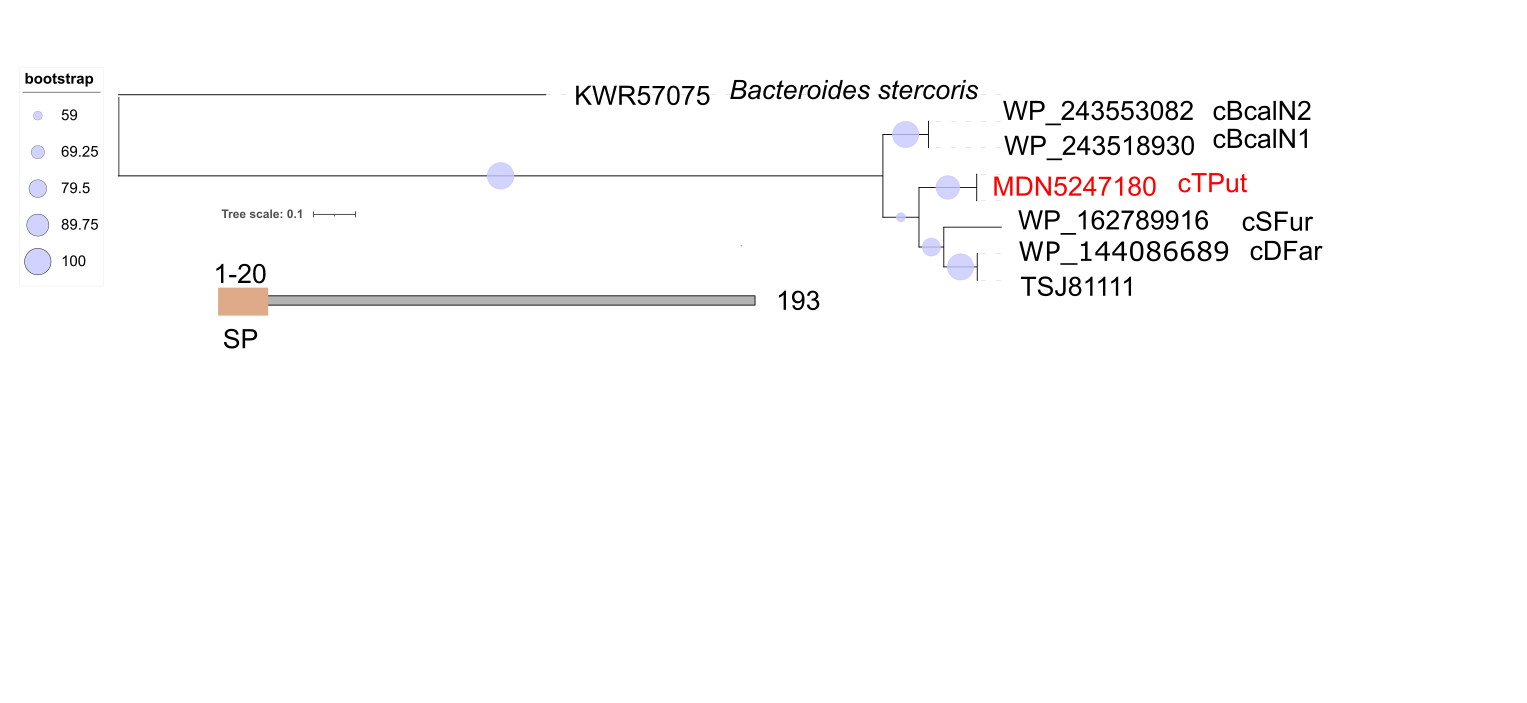


Note: SP - signal peptide

**
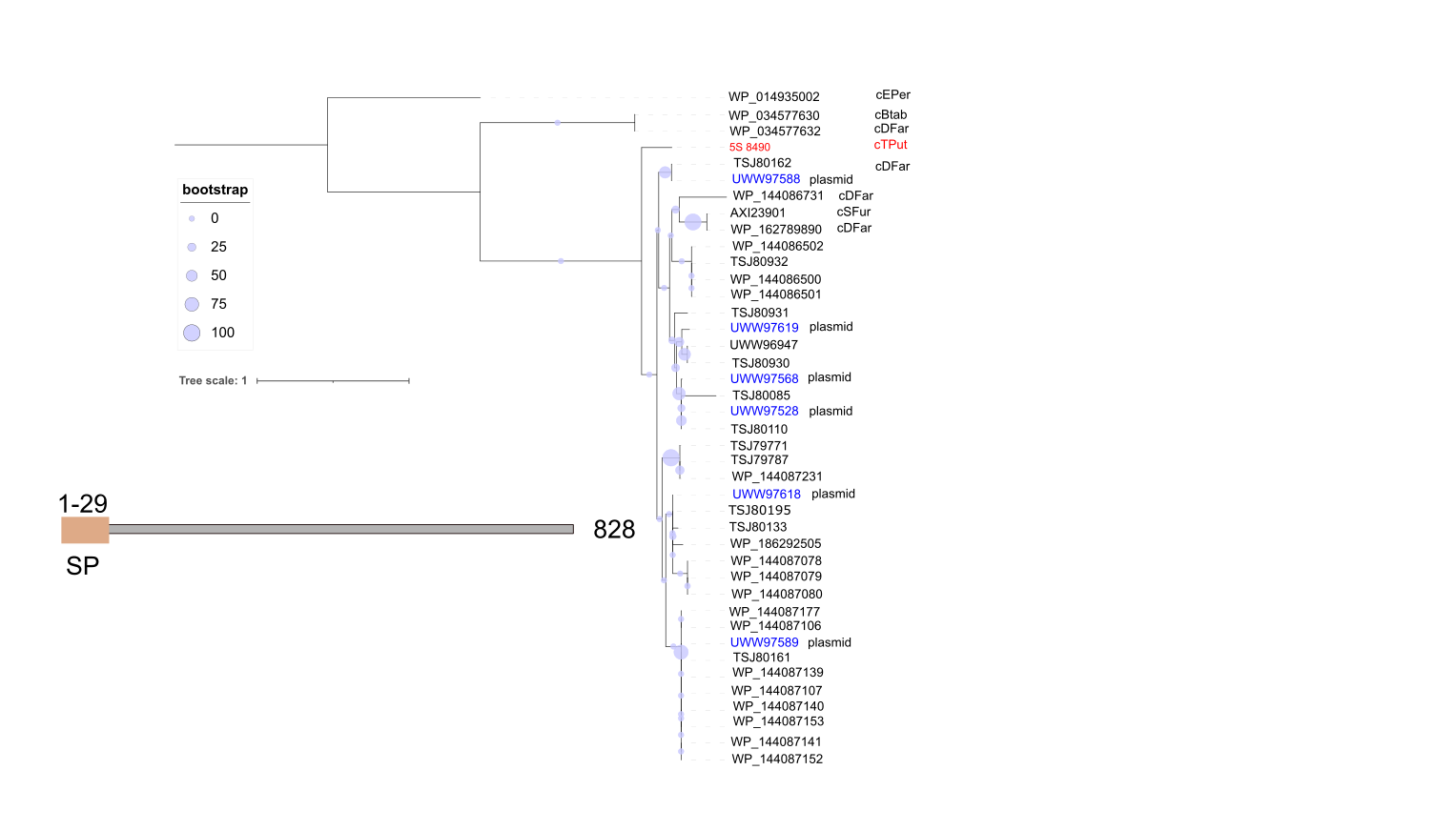
FIG S11** Identification of the *Cardinium* 828-amino acid hypothetical protein (5S_8490, red color) based on GenBank data. Both unrooted tree and HMMER (101) model are shown. Blue color indicates cDFar plasmid located proteins.

Note: SP - signal peptide

**FIG S12** Identification of the *Cardinium* Type IV secretion-system coupling protein DNA-binding domain (5S_8750 242 aa, 5S_8460 6634aa, and 5S_7750 630 aa) using GenBank data. The tree was rotted to *Flavobacterium psychophilum* (Genbank accession CAL42593). The HMMER (101) models are shown. The red terminal text label color indicates cTPut proteins, while blue color indicates plasmid associated proteins. The triangles in the HMMER (101) models indicate non-aligned start and/or end of the protein.
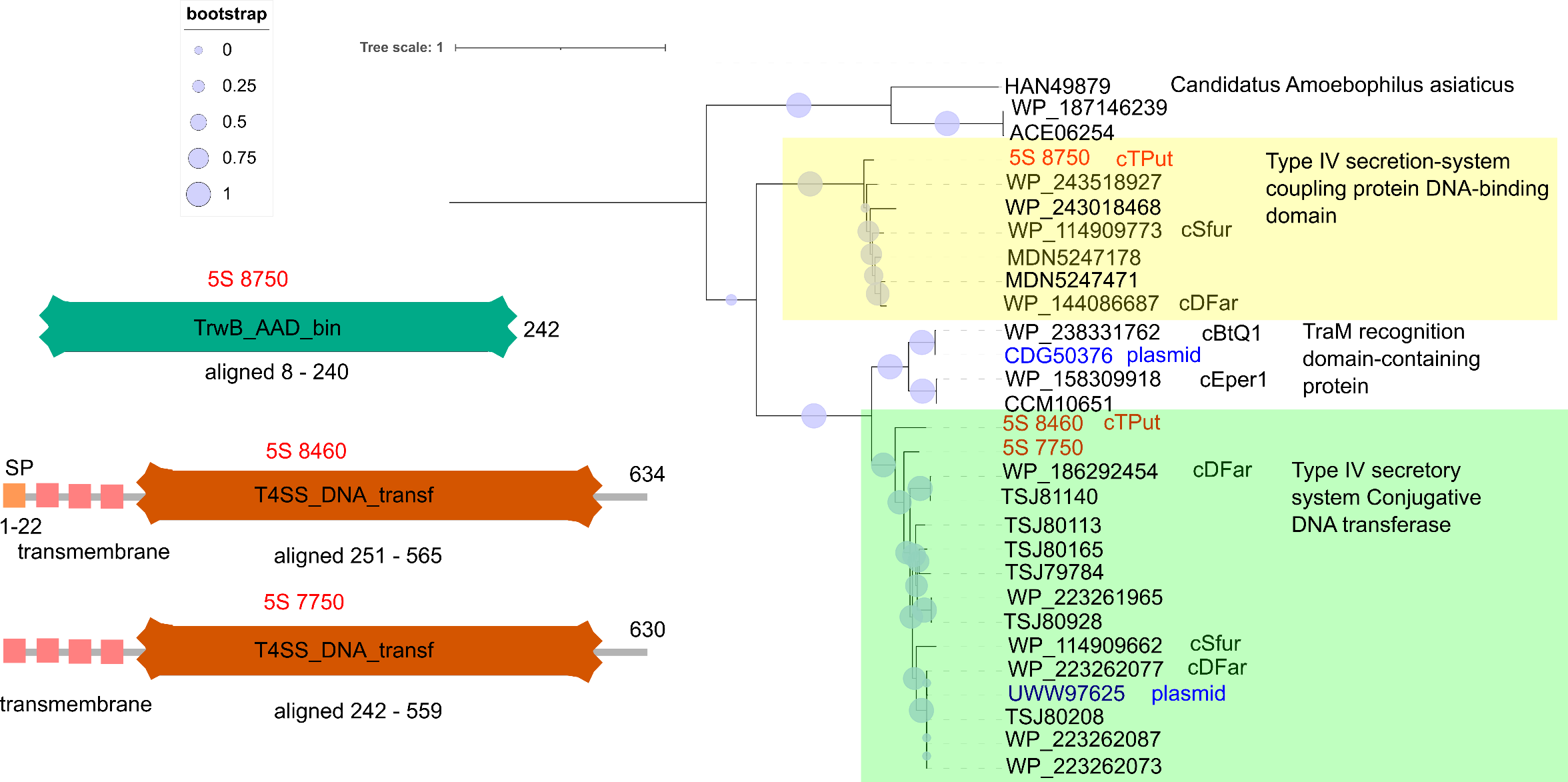


Note: SP – signal peptide

**FIG S13** Correlation network for *Cardinium* (cTPut) hypothetical protein (GenBank id: WP_260536282) and *Tyrophagus putrescentiae* immune and KEGG regulatory protein expression; **A** – Network based on the samples when *Erwiniaceae* symbiont is absent (SLS−) and **B**
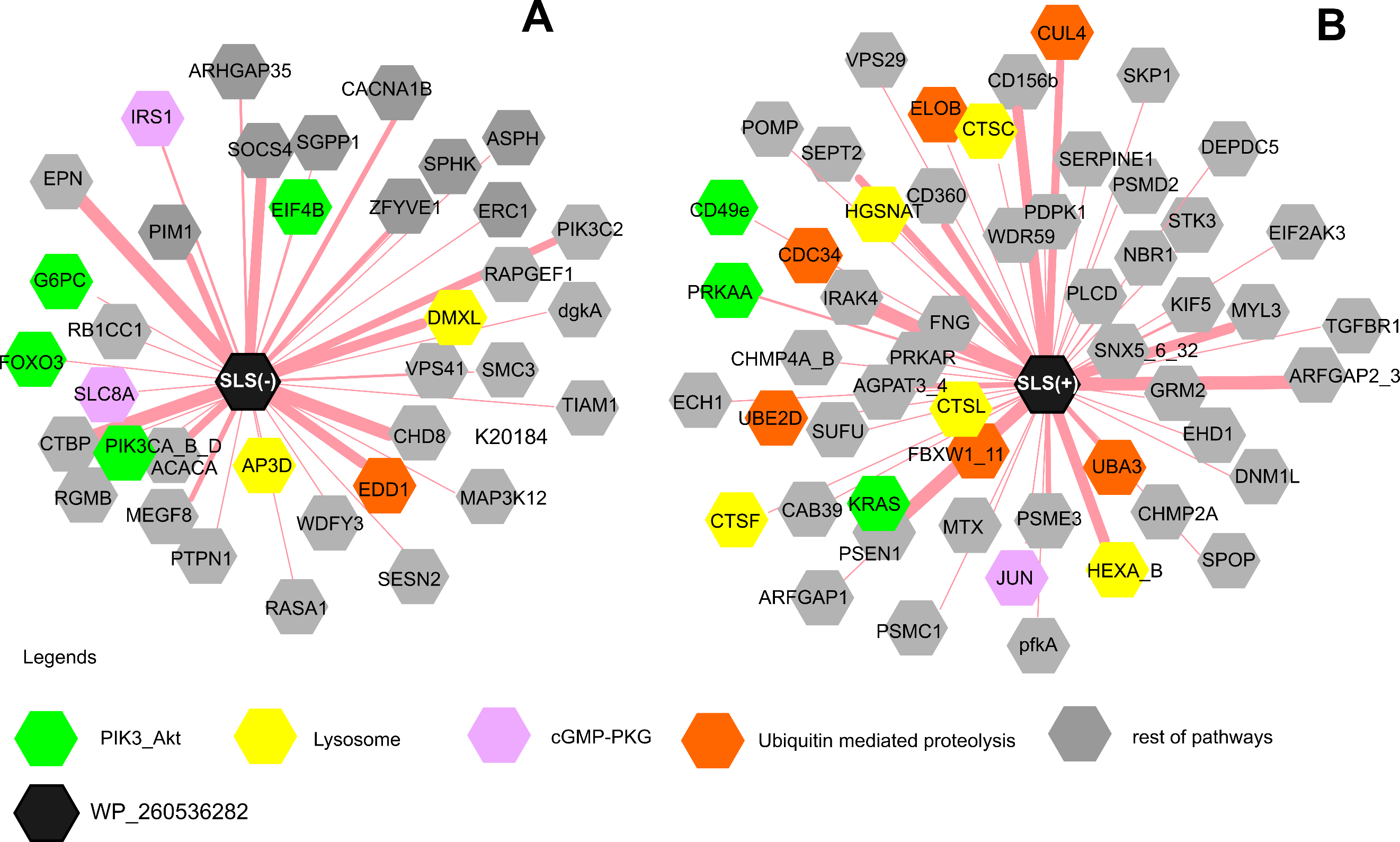
present (SLS+).

Note: The Cytoscape networks are based on absolute Spearman correlation coefficients (0.75 to 1.00, permutational P < 0.05). Positive correlations are shown in red, and negative correlations are shown in blue.


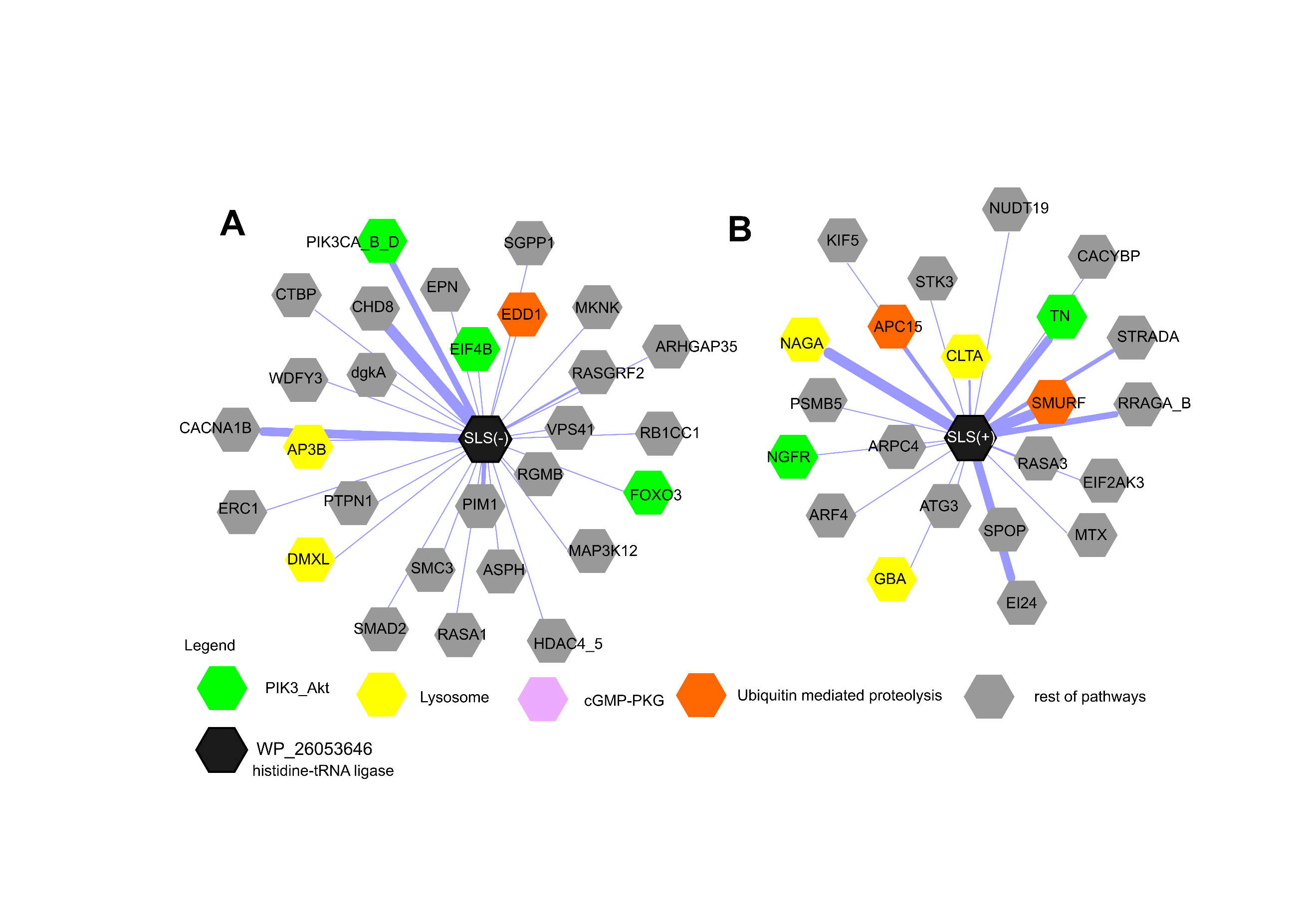
**FIG S14** Correlation network for *Cardinium* (cTPut) histidine-tRNA ligase (GenBank Access No WP_26053646) and *Tyrophagus putrescentiae* immune and KEGG regulatory protein expression; **A** – Network when *Erwiniaceae* symbiont is absent (SLS−) and **B** present (SLS+).

**Note:** The Cytoscape networks are based on absolute Spearman correlation coefficients (0.75 to 1.00, permutational P < 0.05). Positive correlations are shown in red, and negative correlations are shown in blue.


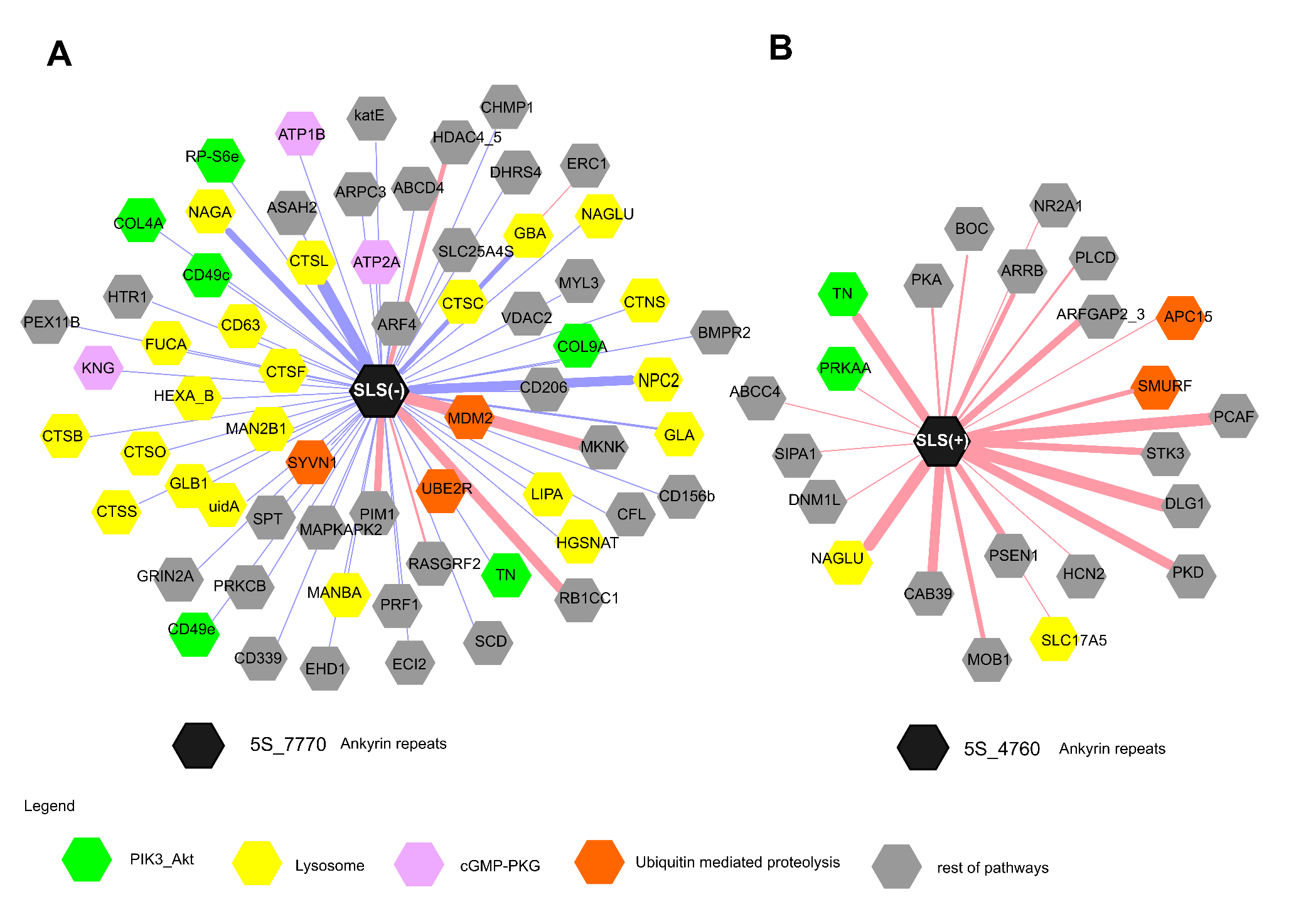
**FIG S15** Correlation network for *Cardinium* (cTPut) ankyrin containing proteins and *Tyrophagus putrescentiae* immune and KEGG regulatory protein expression; **A** – 5S_7770 in the network based on the samples without *Erwiniaceae* symbiont (SLS−) and **B** – protein (GenBank Access No WP_260536329) in the network based on the samples with *Erwiniaceae* symbionts (SLS+).

**Note** The networks are based on absolute Spearman correlation coefficients (0.75 to 1.00, permutational P < 0.05). Positive correlations are shown in red, and negative correlations are shown in blue.


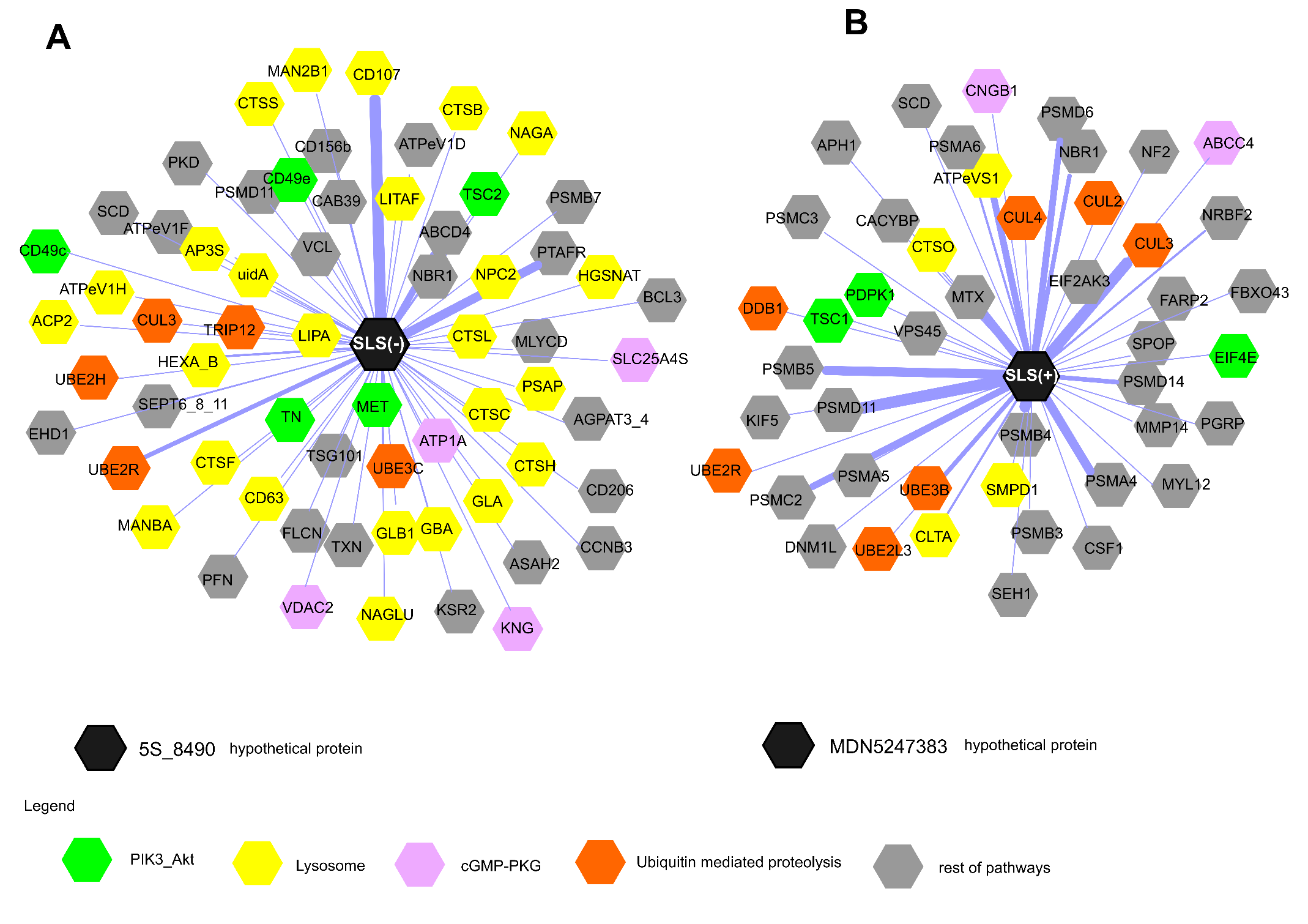
**FIG S16** Correlation network for *Cardinium* (cTPut) hypothetical proteins and *Tyrophagus putrescentiae* immune and KEGG regulatory protein expression; **A** – 5S_8490 in network based on samples without *Erwiniaceae* symbiont (SLS−), and **B** – *Cardinium* protein (GenBank Access No MDN5247383) in the network based on the samples with *Erwiniaceae* symbiont (SLS+).

**Note:** The networks are based on absolute Spearman correlation coefficients (0.75 to 1.00, permutational P < 0.05). Positive correlations are shown in red, and negative correlations are shown in blue.


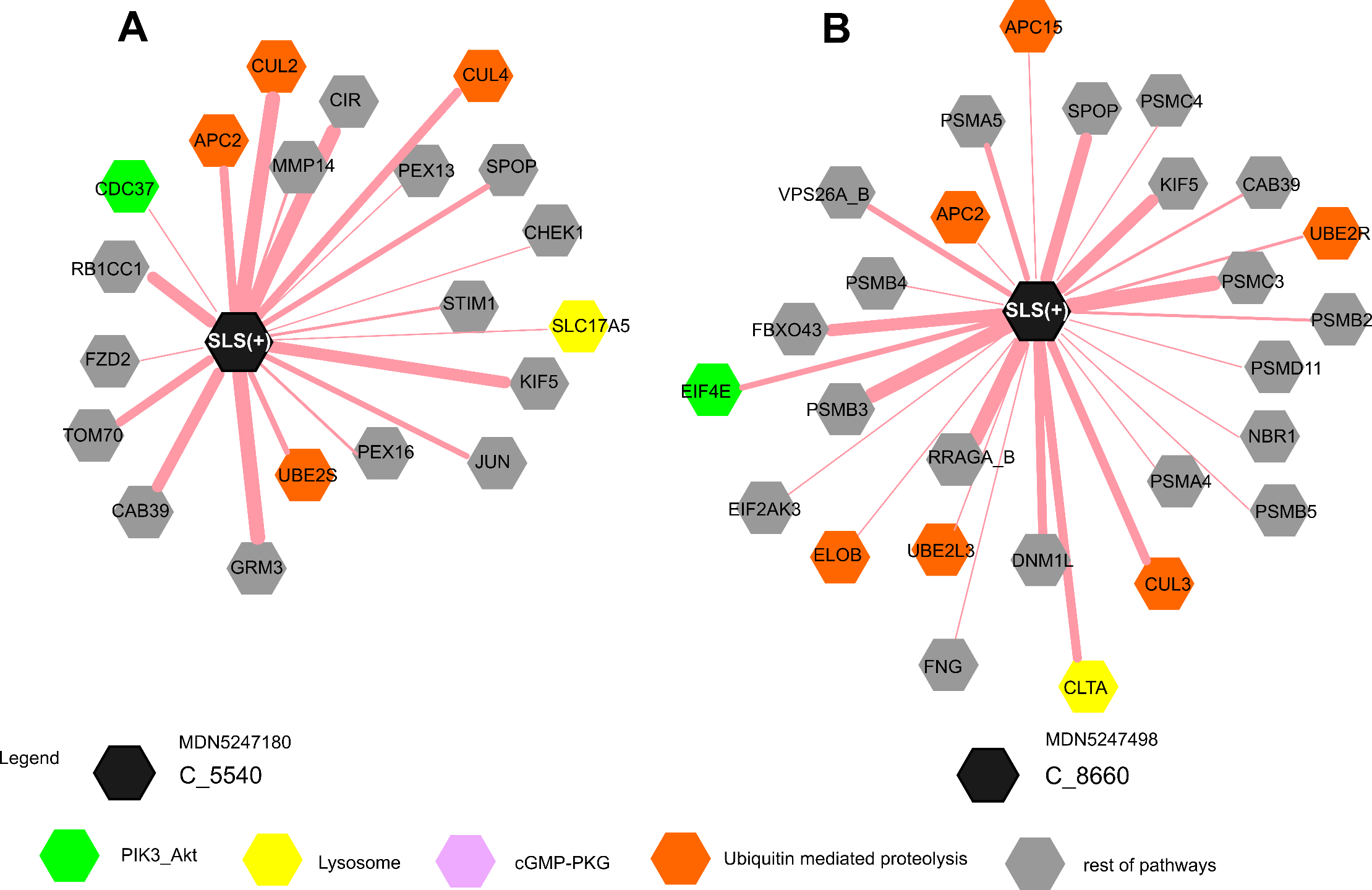
**FIG S17** Correlation networks for two hypothetical *Cardinium* (cTPut) protein expression and *Tyrophagus putrescentiae* immune and KEGG regulatory protein expression in the samples with *Erwiniaceae* symbionr (SLS+). **A** – C_5540 protein (GenBank ID: MDN5247180) and **B** – C_8660 protein (GenBank ID: MDN5247498).

**Note:** The networks are based on absolute Spearman correlation coefficients (0.75 to 1.00, permutational P < 0.05). Positive correlations are shown in red, and negative correlations are shown in blue.


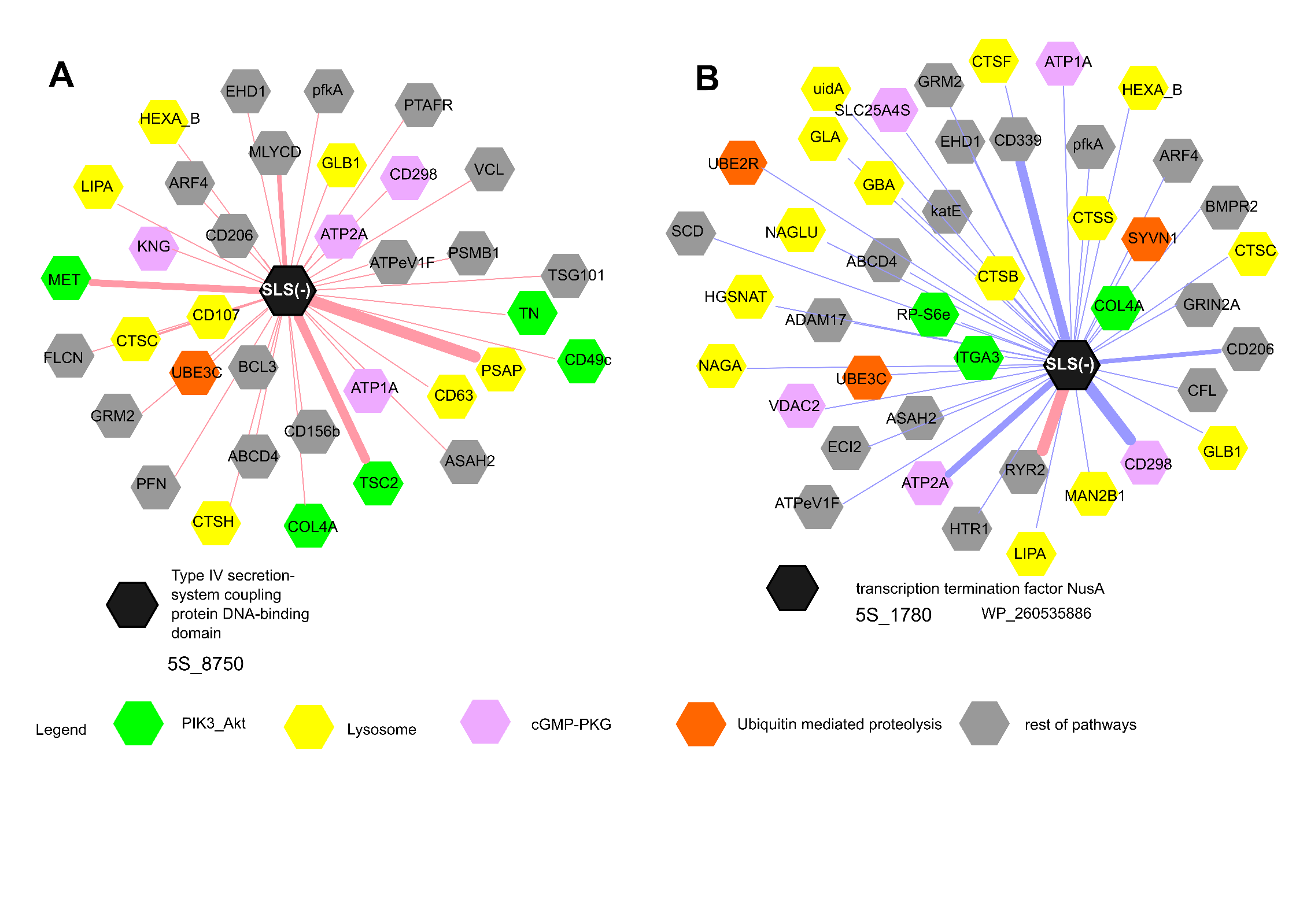
**FIG S18** Correlation networks for two *Cardinium* (cTPut) gene expression and *Tyrophagus putrescentiae* immune and KEGG regulatory protein in the samples without *Erwiniaceae* symbiont (SLS−). **A** – 5S_8750 Type IV secretion-system coupling protein DNA-binding domain and **B** – 5S_1780 transcription termination factor NusA (GenBank id :WP_260535886).

Note Cytoscape networks are based on absolute Spearman correlation coefficients (0.75 to 1.00, permutational P < 0.05). Positive correlations are shown in red, and negative correlations are shown in blue.


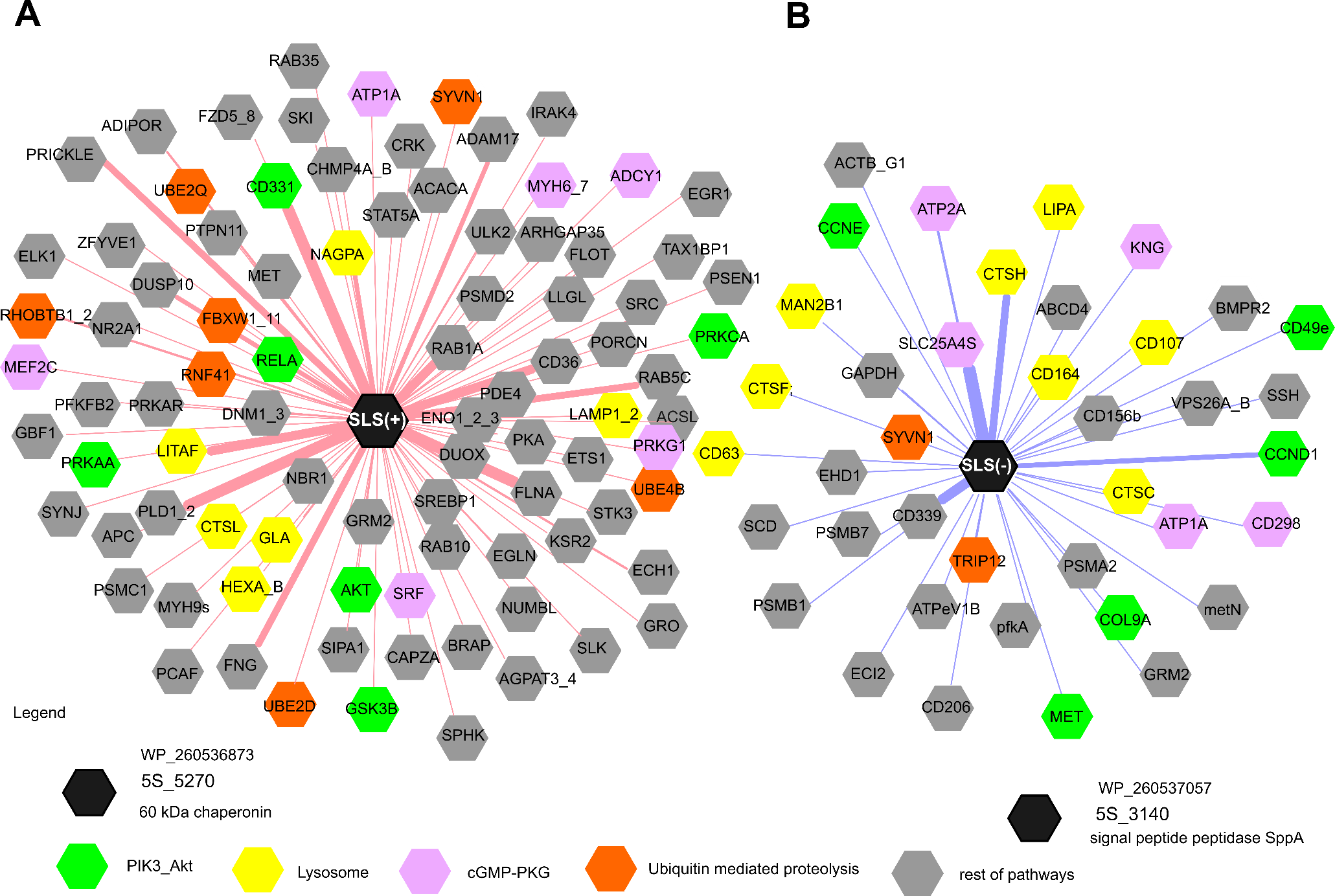
**FIG S19** Correlation networks for two *Cardinium* (cTPut) and *Tyrophagus putrescentiae* immune and KEGG regulatory protein; **A** – 5S_5270 60 kDa chaperonin (GenBank id: WP_260536873) protein in the samples without *Erwiniaceae* symbiont (SLS-); and **B** – 5S_3140 signal peptide peptidase SppA (GenBank id: WP_260537057) in the samples with *Erwiniaceae* symbiont (SLS+).

Note Cytoscape networks are based on absolute Spearman correlation coefficients (0.75 to 1.00, permutational P < 0.05). Positive correlations are shown in red, and negative correlations are shown in blue.

**SUPPLEMENTARY REFERENCES**

43. Olm MR, Brown CT, Brooks B, Banfield JF. 2017. dRep: a tool for fast and accurate genomic comparisons that enables improved genome recovery from metagenomes through de-replication. ISME J 11(12):2864–2868. <https://doi.org/10.1038/ismej.2017.126>

101. Eddy SR. 2011. Accelerated profile HMM searches. PLoS Comput Biol 7(10):e1002195. <https://doi.org/10.1371/journal.pcbi.1002195>

103. Galaxy Community. 2024. The Galaxy platform for accessible, reproducible, and collaborative data analyses: 2024 update. Nucleic Acids Res 52(W1):W83–W94. <https://doi.org/10.1093/nar/gkae410>
